# Scanning Drop Friction Force Microscopy

**DOI:** 10.1021/acs.langmuir.2c02046

**Published:** 2022-11-18

**Authors:** Chirag Hinduja, Alexandre Laroche, Sajjad Shumaly, Yujiao Wang, Doris Vollmer, Hans-Jürgen Butt, Rüdiger Berger

**Affiliations:** † Max Planck Institute for Polymer Research, Mainz 55128, Germany; ‡ University of Zurich, Winterthurerstrasse 190, Zurich 8057, Switzerland; § Key Laboratory of Interfacial Physics and Technology, Shanghai Institute of Applied Physics, Chinese Academy of Sciences, Shanghai 201800, China; ∥ University of Chinese Academy of Sciences, Beijing 100049, China

## Abstract

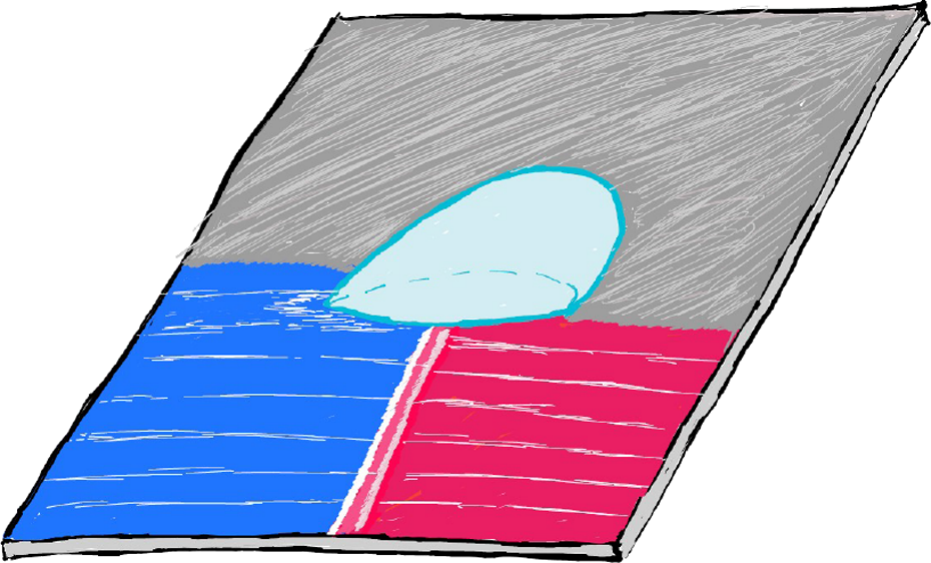

Wetting imperfections are omnipresent on surfaces. They cause contact angle hysteresis and determine the wetting dynamics. Still, existing techniques (e.g., contact angle goniometry) are not sufficient to localize inhomogeneities and image wetting variations. We overcome these limitations through scanning drop friction force microscopy (sDoFFI). In sDoFFI, a 15 μL drop of Milli-Q water is raster-scanned over a surface. The friction force (lateral adhesion force) acting on the moving contact line is plotted against the drop position. Using sDoFFI, we obtained 2D wetting maps of the samples having sizes in the order of several square centimeters. We mapped areas with distinct wetting properties such as those present on a natural surface (e.g., a rose petal), a technically relevant superhydrophobic surface (e.g., Glaco paint), and an in-house prepared model of inhomogeneous surfaces featuring defined areas with low and high contact angle hysteresis. sDoFFI detects features that are smaller than 0.5 mm in size. Furthermore, we quantified the sliding behavior of drops across the boundary separating areas with different contact angles on the model sample. The sliding of a drop across this transition line follows a characteristic stick–slip motion. We use the variation in force signals, advancing and receding contact line velocities, and advancing and receding contact angles to identify zones of stick and slip. When scanning the drop from low to high contact angle hysteresis, the drop undergoes a stick–slip–stick–slip motion at the interline. Sliding from high to low contact angle hysteresis is characterized by the slip–stick–slip motion. The sDoFFI is a new tool for 2D characterization of wetting properties, which is applicable to laboratory-based samples but also characterizes biological and commercial surfaces.

## Introduction

Nonwetting coatings find a place in a variety of industrial applications, for instance, dirt-repellent surfaces,^1^ microfluidics,^2^ anti-icing, and antifogging surfaces,^3^ to name a few.^2−6^ For their optimum performance, it is important to have a homogeneous hydrophobic coating. However, in reality, failure in the coating preparation process or harsh ambient conditions result in nonuniform or degraded coatings.^7^ Undesired localized variations in the wetting properties appear due to topographical or chemical surface imperfections. These localized variations hinder the sliding motion of the drops. Often the location and distribution of these localized imperfections on the surfaces are unknown. Therefore, we have developed the scanning drop friction force microscopy (sDoFFI) technique. This technique allows us to image, localize, and characterize the variations in wetting properties down to the submillimeter scale.

Contact angle (CA) measurements of sessile drops serve as a standard method for the characterization of the surface wetting properties, along with the determination of the roll-off angle.^8,9^ In sessile drop measurements, the initial advancing drop volume to measure the receding CA depends critically on surface properties, like whether it is hydrophilic, hydrophobic, or superhydrophobic.^10^ For instance, on surfaces with a high CA hysteresis (CAH = θ_a_ – θ_r_), the drop volume may span from 5 to 100 μL to measure the advancing contact angle (θ_a_) and the receding contact angle (θ_r_) accurately. Measurements corresponding to the lowest volume of the sessile drop, that is, 5 μL, could cover a contact area of ≈4 mm^2^. Hence, any details smaller than the contact area are likely to be missed.^11^ In addition, the sessile drop method is a point-based approach. Samples having areas of 50 × 50 mm^2^ need to be discretized into a handful of points where localized CA measurements would be performed. On average, each localized CA sessile drop measurement entails 3–5 min of data recording. Consequently, a large area characterization would entail 1–3 h. Furthermore, the CA determination is susceptible to the positioning of a baseline and optical resolution of the camera.^10,12−14^

In the recent past, attempts have been made to overcome these limitations by force-based measurement techniques.^15−18^ For instance, vertical drop adhesion force^16,18^ and viscous dissipation^17^ have come up as potential techniques. The former technique works on the principle of CA measurements and is a point-based approach.^19^ This technique offers a spatial resolution of 10 μm and a force resolution in the nN range and is useful for mapping very small-scale wetting variations on superhydrophobic surfaces. The latter technique is limited to superhydrophobic surfaces with almost no CA hysteresis. In addition, the existing techniques fall short in determining the distribution of the inhomogeneities in the centimeter scale.

**Figure 1:**
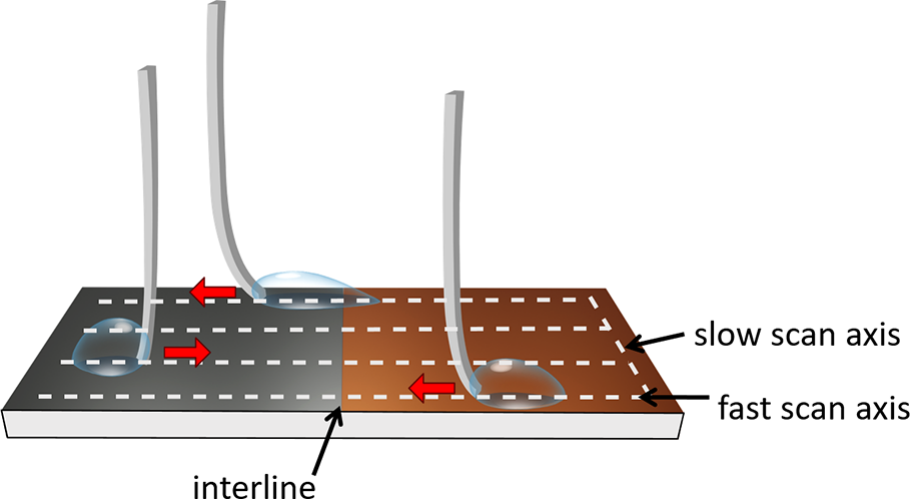
Schematic representation of sDoFFI. A drop is attached to an elastic spring and is raster-scanned relative to the sample in two dimensions. The drop slides relative to the sample from one area (brown color) to the adjacent area (gray color). The dashed gray lines represent the trace of the sliding drop. The red arrow is the relative slide direction. The line shift (ls) is in the order of the width of the drop or even smaller on-demand. In this representation, the brown area corresponds to a surface with higher CA hysteresis compared to the gray one. Accordingly, the elastic spring bends more on the brown area due to a higher capillary force. In the transition area (at the interline), the CAs vary and stick–slip phenomena of the contact line occur.

To overcome these limitations, we extend the friction (lateral drop adhesion) force measurements^20,21^ into a 2-dimensional surface characterization tool—sDoFFI (Figure 1). This new technique localizes and characterizes the variations of wetting properties on surfaces. We raster-scan a liquid drop over a surface and measure the force required to move the drop. The wetting images are obtained by plotting the friction force (*F*_DoFFI_) versus the position of the drop on the sample. The capillary force (*F*_cap_) acting on a sliding drop is:^22−28^
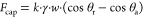
1where *w* is the width of the drop, γ is the liquid surface tension, and *k* is a geometrical factor accounting for the drop shape. Equation 1 is used to calculate the retention force on the drop on an inclined surface. The conditions for this equation are a defined mathematical shape (circular^24^ or parallel sided^25^ or elliptical^27,28^) of the base contour and distribution of the CA (constant, linear, cosine or polynomial) from the advancing to receding ends. Based on the drop’s base contour and distribution of CA assumed in the derivation, different values of “*k*” have been reported. Krasovitski and Marmur pointed out that the CAs determined by a tilted plate setup do not in general equal the advancing and receding CAs.^29^ Therefore, using eq 1 for calculating the friction force on drops can be associated with an error for hydrophobic surfaces with large CA hysteresis. Thus, by measuring the sliding force of drops, assuming *F*_DoFFI_ ≈ *F*_cap_, different wetting properties can be analyzed.^30−33^ In other words, the direct dependence of the capillary force on these CAs paves a way for avoiding standard CA goniometry for surface wettability characterization.

Using sDoFFI, we investigate the force signals emanating when the three-phase contact line moves over an inhomogeneous surface. The force signals arise from variations in surface properties according to eq 1, and the water drop acts as a probe for determining the wetting differences. The developed scanning technique for the characterization of surface wetting properties is an elegant way to overcome the temporal and local resolution limitations posed by standard CA goniometry or by existing scanning droplet adhesion force microscopy. We divide our study into two parts: In part A, we investigate how a drop slides over an abrupt wetting transition. We elucidate the drop sliding behavior and quantify the friction forces acting on the advancing and receding parts of the contact line when it transitions across the interline. In part B, we apply the sDoFFI method to different samples, ranging from technologically relevant to natural surfaces.

## Experimental Procedures

We aim for a surface with two distinct regions of CA hysteresis. CA hysteresis can arise out of roughness or surface chemistry change. Both lead to changes in the friction force. Here, we achieve a distinct surface chemistry by applying a double chemical vapor deposition process. We achieve roughness change by preparing a surface coated with two different layers of Glaco (mirror coat zero). At last, we also study the back side of a rose petal, which constitutes friction force due to both roughness and chemical changes.

### Sample Preparation Methodology

#### PFOTS Deposition

**Figure 2:**
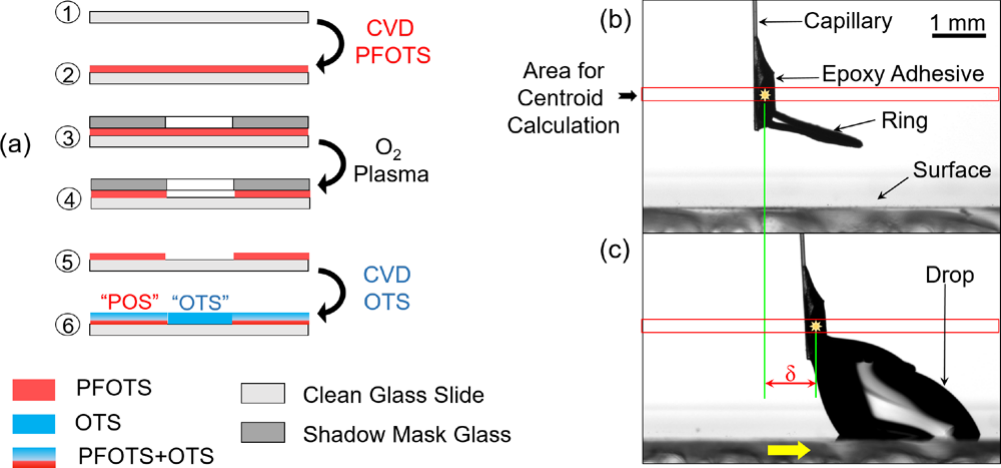
(a) Schematic representation of the sample preparation procedure. Step 1 shows a plasma-cleaned glass slide (gray); step 2, a PFOTS coating is present (red); step 3, clean glass slides are used as a mask (dark gray), and then the assembly is plasma cleaned in step 4, removing unmasked PFOTS; step 5, the mask is removed; and finally, in step 6, we obtain a patterned surface made with OTS molecules (blue region) and combined PFOTS + OTS molecules (POS, red-blue region). (b) Side view of the end of the glass capillary where a metal ring is attached. The end of the glass capillary is 2 mm away from the surface. (c) The metal ring holds a water drop and the substrate underneath is moved to the right side. The substrate movement is indicated by an arrow and here the drop is pulled. We call this configuration “forward motion”, and we term the opposite stage motion as “reverse motion”. In response to the forward motion of the stage, the glass capillary bends to the right by δ, in this case, 1.1 mm. The red lines presented here are used for selecting the image area at which deflection is measured. The yellow dot represents the centroid of the dark region confined within the red lines. The green lines show the deflection of the capillary from the undeflected position, caused by the friction force.

Standard microscopic glass slides (75.9 mm × 25.7 mm) are thoroughly cleaned first with Milli-Q water and then with acetone and ethanol followed by O_2_-plasma treatment for 5 min (300 W at 0.3 bar, Diener Electronic Femto). These plasma-treated glass slides (step 1 in Figure 2a) are immediately placed in a desiccator with 1 mL of trichloro(1*H*,1*H*,2*H*,2*H*–perfluorooctyl)silane (PFOTS, Sigma-Aldrich, quality 97%) and a low-pressure atmosphere (<20 mbar). The low-pressure condition is maintained by continuously pumping air out of the chamber. After 10 min, the pump is switched off. The samples remain in the chamber at a low pressure for the next 20 min (step 2 in Figure 2a). The obtained glass slides are then immediately transferred to a vacuum oven tempered at ambient temperature for 10 min. The prepared samples are finally stored in a closed Petri dish for 1 day before use.

#### OTS Deposition

The PFOTS samples obtained using the abovementioned steps are covered with a shadow mask made from glass (step 3 in Figure 2a) to create the desired patterns. The covered glass slides are then kept in the O_2_-plasma chamber for 5 min (300 W, 0.3 bar). With this exposure, we obliterate the PFOTS layer from the unmasked portion (step 4 in Figure 2a). After plasma treatment, the shadow mask is removed (step 5 in Figure 2a) and the samples are immediately placed in the desiccator with 200 μL of octyltrichlorosilane (OTS, Sigma-Aldrich, quality 97%) for chemical vapor deposition (CVD). We wait until the vacuum condition (150 mbar) is reached. Once reached, we close the desiccator valve and disconnect the vacuum pump. The samples remain in vacuum for the next 120 min (step 6 in Figure 2a). The obtained patterned samples are rinsed with Milli-Q water before use. This procedure results in a surface partly coated with a layer of only OTS and another part with both PFOTS and OTS (step 6 in Figure 2a). As a convention, we use red color for the PFOTS and OTS surface (in short, POS). For the OTS surface, we use blue color (Figure 2a). The double CVD procedure we followed here is not common. A more detailed analysis is required for understanding the molecular structure resulting from this CVD procedure.

### CA Measurements

#### Sessile Drop Method (Goniometer)

The sample obtained after the final preparation step is characterized by standard CA goniometry. To calculate the CA hysteresis on the obtained sample, we use a Krüss DSA100 goniometer. For advancing CA measurements on both surfaces, an initial 3 μL volume of Milli-Q water is deposited on the sample, then the needle and baseline are adjusted, accordingly. Then, we inflate the drop at a volume flow rate of 0.5 μL/s until the total drop volume of 22 μL is reached (Figure S1a,c). For receding CA measurements, an initial drop volume of 45 and 70 μL is deposited on OTS and POS, respectively. Then, the drop is deflated at the same volume flow rate. To calculate the CAs, we use the tangent fit method provided by the software (Figure S1b,d). The CAs are measured at three different locations on each surface chemistry side on a single sample. The set of data in which the contact line started to move is presented in the plots (Figure S1). The θ_a_ and θ_r_ values on POS area are 128° ± 2° and 72° ± 2°, respectively (Figure S1). However, we could not reach a complete plateau on the receding side. In the areas with only OTS, we measure θ_a_ of 113° ± 2° and θ_r_ of 89° ± 2°.

#### Drop Sliding

We also calculate the advancing and receding CAs when the drop actually slides on the surface. We determine CAs from the same image data from which the deflection of the capillary sensor is quantified. We analyze the CAs from the obtained video data, frame-by-frame, using a python script. To calculate CAs, we carefully mark the baseline within the code and use the tangent fit. For fitting a tangent to the edges, 20 pixels on each edge (image resolution ≈ 10 μm/px) are considered. The θ_a_ and θ_r_ values across six different samples on the POS area are found to be 124° ± 5° and 61° ± 4°, respectively. Only on the OTS area, we calculate θ_a_ of 109° ± 2° and θ_r_ of 76° ± 7°.

We observe a difference in measured CA values in both measurement procedures. We attribute this difference to the variation in surface wetting properties across the samples and between samples that are prepared at different days and measured after different times (Figure S1e,f).

### Sensor Calibration

Hollow rectangular glass capillaries (0.05 mm × 0.5 mm × 50 mm, CM Scientific Ltd.) are used as springs for force sensing. This capillary is entrenched in a brass holder with the help of an adhesive (UHU Plus Endfest 2 K Epoxy Adhesive). The length of the capillary protruding out of the holder ranges from 45 to 47 mm. To determine the spring constant, an initial gentle displacement is imparted on the free end of the capillary, and then it is released. The subsequent oscillations of the free end are recorded using a CMOS camera (Figure S2). The measured frequency corresponds to the first fundamental natural frequency ω_*n*_. The spring constant of the hollow rectangular glass capillary κ is then:
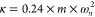
2where *m* is the mass of the capillary protruding out of the base support. The measured spring constant for the capillary is typically in the order of 100 μN/mm with an uncertainty less than 5%. The obtained spring constant is cross-checked with another measurement technique reported by Gao et al.^21^ (Figure S3). The difference between κ values obtained by these two measurement techniques is less than 3%. To immobilize the drop using a glass capillary, we use a metal ring with a diameter of ≈2 mm formed by a wire of a diameter of 0.2 mm. This ring is attached to the end of the glass capillary with the help of adhesive glue (Figure 2b). The attachment of the ring changed the mass at the end of the capillary, but the spring constant remains unaltered. In particular, the ring is well suited for scanning hydrophobic surfaces and surfaces with high CA hysteresis since it keeps the drop in place for scanning samples in the *X*–*Y* direction. Using a ring of ≈14 mm diameter, we are able to slide drop volumes up to 175 μL over a hydrophobic surface (PFOTS on glass) without any detachment from the sensor. In this case, we measure friction force values >600 μN. This obtained force value is almost 4 times higher than the values reported in the results section of the study. In addition, this obtained value is not the limiting value of the friction force; therefore, concerns associated with drop detachment do not find significance here. For force measurements on superhydrophobic surfaces, the ring is not necessary. In the latter case, the adhesion of the water drop to the glass capillary is strong enough to hold the drop in place during scanning.

### Scanning Procedure

Samples are scanned using a computer-controlled XY stage (Krüss DSA100). The stage speed *v*_stage_ in both directions is kept constant at 4 mm/s, unless stated otherwise. We carefully position a 15 μL water drop onto the surface using a micropipette. The drop is then held in position by the metal ring of the glass capillary sensor (Figure 2c). Following this, the stage underneath is moved back and forth in one direction (e.g., *x*-direction) covering the entire length of the sample (fast scan axis). During this motion, the sliding force (through capillary deflection) is recorded and plotted versus drop position. We term this scanned strip as a single “scan line”. Once the drop returns to the initial position of the respective scan line, it makes a shift in the perpendicular direction (e.g., *y*-direction) by a distance less than or equal to the width of the drop. Subsequently, the next scan line is recorded (Figure 1). This process continues until the entire surface is scanned. Each scan line and its associated forward and reverse motions are recorded in separate video files (Supporting Information (SI), Section 3).

## Results and Discussion

### Part A: Drop Sliding across an Abrupt Wetting Transition

**Figure 3:**
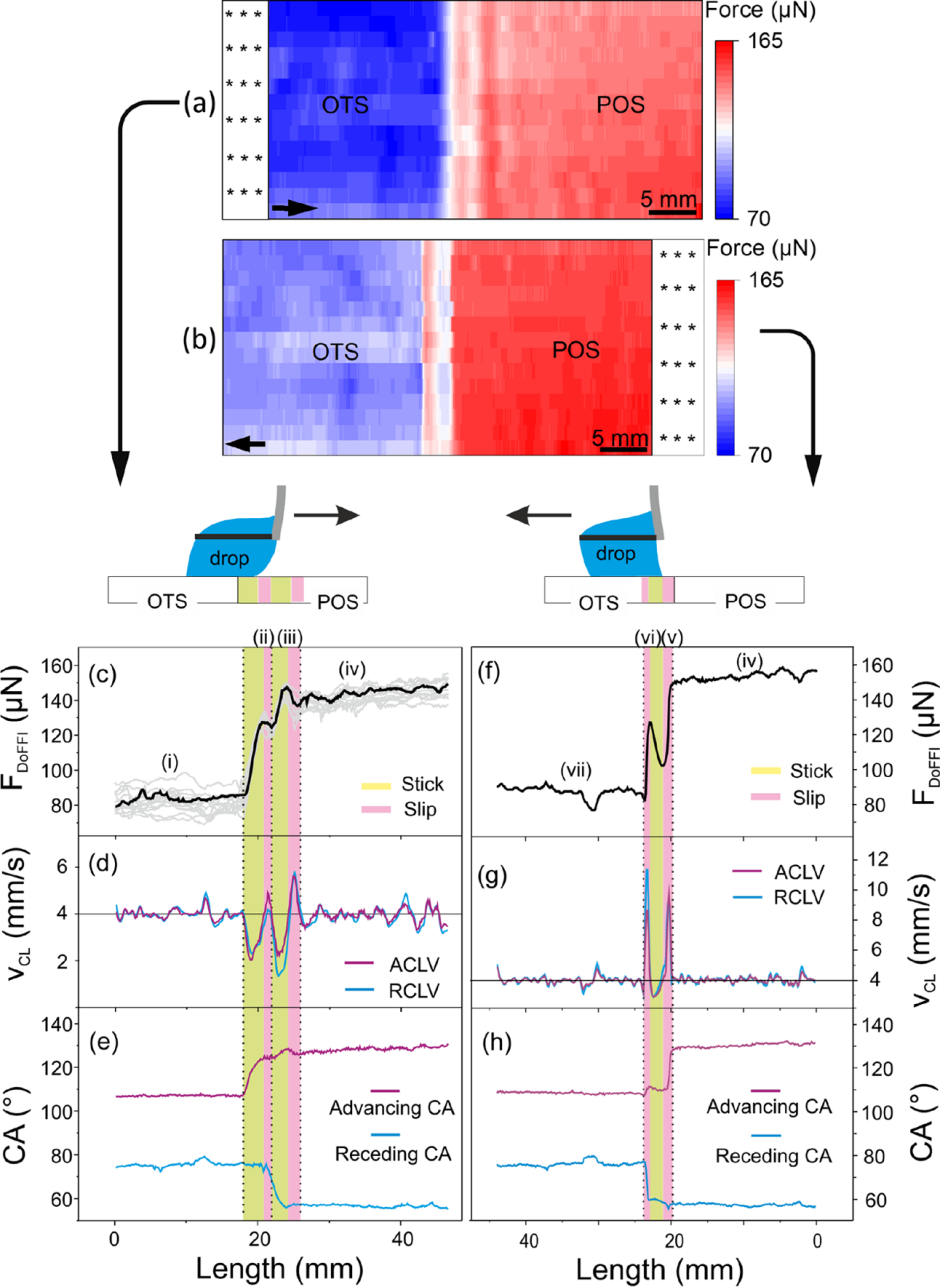
sDoFFI images of the model surface. (a) Forward force map (drop pulling) with two distinct wetting areas, blue (OTS) and red (POS). A water drop of 15 μL volume is used and the line shift is 1.5 mm. The bottom scan line is the first scan line and the top is the last. In total, 14 scan lines have been used for these wetting maps. The force occurring in the static regime is discarded (dotted area) and only the force while sliding is used (Section 4, Supporting Information). (b) Reverse force map is recorded while pushing the drop. (c) Representative force profile along one of the scan lines of the forward force map in panel (a). All the other force profiles are plotted as gray lines. (d) Velocity profile of the advancing and receding contact line (ACLV and RCLV) in the forward motion for the scan line shown in the force profile. (e) CAs along the forward scan line. (f) Force profile in the reverse direction along one of the scan lines. (g) Advancing and receding contact line velocities along the reverse scan line. (h) CAs along the reverse scan line.

We scan a model surface having two chemically distinct regions with different CA hysteresis (Figure 2a). This surface has a portion with a layer of OTS and a layer of PFOTS/OTS (“POS”) with an abrupt transition to a portion with only OTS. The POS surface has a CA hysteresis of ≈60° that decreases to ≈25° for OTS. Mapping such a surface over an area of 55 × 22 mm^2^ by sDoFFI reveals two distinct force values (enclosed areas in Figure 3a). These force values are represented by a blue-to-red color scale. Analyzing these areas reveal a mean force of *F*_OTS_ = 81.5 μN ± 5.5 μN and *F*_POS_ = 143 μN ± 4.5 μN for the OTS and POS areas, respectively. At the OTS to POS transition, additional force signals appear when the drop transitions across the interline.

#### Stick–Slip–Sliding

The transition of a sliding drop from the OTS to the POS surface is characterized by an increase in *F*_DoFFI_ from *F*_OTS_ ≈ 86 μN to a first local maximum (≈128 μN) followed by second global maximum in force *F*_DAFI_ ≈ 148 μN and finally to *F*_POS_ ≈ 144 μN (Figure 3c (i–iv)). Both maxima persist for all scan lines at an almost identical position (Figure 3c—overlay of gray scan lines). Analyzing the drop velocity from videos of the side camera indicates that the advancing and receding contact line velocities vary at the transition line (Figure 3d). We use the advancing and receding contact line velocities to distinguish between zones where the drop sticks (drop velocity < stage velocity of 4 mm/s) and drop slips (drop velocity >4 mm/s). This distinction leads to two stick and two slip events, which we attribute to the interactions of the advancing and receding contact line with the OTS–POS interline, respectively. Both stick events are highlighted in yellow and the slip events in red color (Figure 3c–e).

The first stick event corresponds to the pinning of the advancing contact line. *F*_DoFFI_ increases to its first local maximum (≈128 μN). Pinning of the advancing contact line is marked by the gradual increase in θ_a_ from 106 to 124°, while θ_r_ remains constant at 74° (Figure 3e). The minimum velocity of the apex point of the advancing contact line does not coincide with the local maximum in force (Figure 3c,e (ii)). We speculate that the first force maxima correspond to a situation where already part of the advancing side of the drop is in contact with the low energy surface (POS). Notably, after the drop reaches the local maximum in pinning force, it slips for a very short length (red region). This slip process results in a slight dip in the force and θ_r_ starts to decrease. This first stick–slip process corresponds to white and light red pixels, respectively, next to the blue region in the forward force map (Figure 3a).

After the first slip process, the advancing and receding contact line velocities decrease again below the stage velocity (yellow region in Figure 3c–e). Now, the receding contact line interacts with the interline and causes the second pinning of the drop. The θ_r_ value decreases from 74° to 55° while the drop’s force increases to a maximum (Figure 3c, iii). The global maximum force value coincides with the maximum difference between θ_a_ (128°) and θ_r_ (55°). This maximum corresponds to the dark red color band adjacent to the interface in the forward force map (Figure 3a). After the maximum force, both the advancing and receding contact line velocities increase, marking the second slip (red region, Figure 3c). Accordingly, a slight dip in force is noted again, represented by a narrow white-colored region on the immediate right of the dark red area in the forward force map (Figure 3a). Following this second depinning event, the drop continues to slide on the POS at an almost constant force, with a constant θ_a_ of 126° and θ_r_ of 57° (Figure 3c, iv).

The pinning force for the drop sliding from OTS to POS at the interline is highest when the RCL is pinned. Thus, the quantity *w* · ( cos θ_r_ – cos θ_a_) becomes maximal. Pinning of the receding side of a drop corresponds to the elongation of the drop, which by conservation of mass implies a decrease in *w*. Thus, the increase of the term cosθ_r_ – cos θ_a_ dominates the pinning force. The entire transition from OTS to POS follows the “stick–slip–stick–slip” sliding process and covers a length >5 mm. Now, the question arises: are the varying force signals solely from the stick–slip motion or are there any hidden inhomogeneous regions on the POS area in the vicinity of the transition line? To examine the wetting properties of the POS near the interline and in the static regime (dotted area in Figure 3a), we make the drop to retrace the same path in the reverse direction just after completion of the respective forward scan line.

In the reverse direction, the ring is ahead of the capillary and the drop is being pushed. The wetting force map in the reverse direction therefore contains the lost wetting information of the forward scan map (Figure 3b). We record *F*_POS_ = 153 μN ± 3.5 μN and *F*_OTS_ = 98 μN ± 5.3 μN in the POS and OTS areas, respectively. Notably, by doing the retrace, we show that signals are from stick–slip–sliding and no inhomogeneities are located on the POS side in the vicinity of the interline.

#### Slip–Stick–Slip Sliding

The friction force in the reverse direction decreases from 155 μN to 110 μN (Figure 3f). On sliding from POS to OTS, we do not detect pinning of the advancing contact line. At the transition, θ_a_ changes from 128° to 109° (Figure 3h). However, the transition of the receding contact line across the interline is marked by an increase in force to a local maxima of 130 μN followed by an abrupt fall to *F*_OTS_ ≈ 100 μN, which suggests pinning of the receding contact line.

When the advancing front of the drop touches the interline, the drop accelerates (Figure 3g, v). Thus, the drop slips upon reaching the interline (red region). Then, the drop velocity becomes less than the stage velocity (stick). During this stick process, θ_a_ increases slightly from 109° to 111° while θ_r_ decreases from 60° to 58°. This stick situation is correlated with an increase in friction force, highlighted in yellow. At this stage, the rear side of the drop stays in the area with a lower θ_r_. Upon reaching the local force maxima of 130 μN, the drop accelerates again and slips completely across the interline (highlighted in red, (vi)). This process is associated with an increase in contact line velocity and a steep increase in θ_r_. Afterward, *F*_DoFFI_ ≈ 95 μN is obtained in the OTS area (Figure 3f, vii). Note that the local force maximum appears on all the scan lines, which can be seen as a red area sandwiched between blue areas in the corresponding force map (Figure 3b). In conclusion, the transition from POS to OTS follows a “slip–stick–slip” sliding behavior. Overall, abrupt wetting transitions constitute specific friction force profiles arising from three-phase contact line pinning and stick–slip motion of the drop.

### Part B: Application of sDoFFI

#### Repeatability and Accuracy

While recording a wetting map of the OTS/POS sample with a 15 μL drop volume, the force gradually decreased for subsequent scan lines (Figure S8). This decrease is caused by either evaporation of the probing water drop or micron-sized residual drops remaining on the surface. A decreasing drop volume decreases the drop contact width *w* and, according to eq 1, the friction force. Therefore, when scanning a large surface area, the probing water drop needs to be refilled or replaced by a fresh one having the initial volume at the start of the *n*^th^ scan line. The latter is required depending on the scan area, scan speed, and the relative humidity of the ambient environment. Alternatively, a drop of an ionic liquid or a glycerol–water mixture could be employed to slow down the evaporation, depending upon the relative humidity, even stopped.

**Figure 4:**
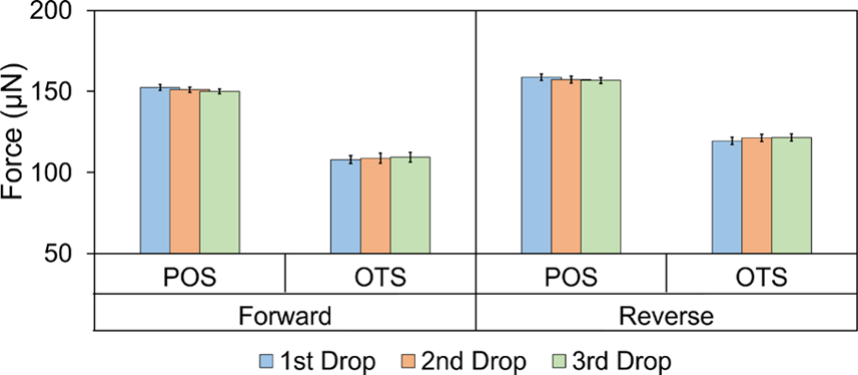
Repeated measurements of the friction force for three different drops on POS and OTS surfaces for forward and reverse directions. The error bar represents three different measurements and the statistical variations within a scan line.

We demonstrate the repeatability of sDoFFI measurements by scanning a single line on the OTS|POS sample with three different drops. Each time, we place a 15 μL drop on the surface, adjust the capillary spring sensor, and measure the sliding force several times (Figure 4). For the forward scan, we measure forces within a 5% variation for drops 1, 2, and 3 on both POS and OTS area. Thus, replacing a probing drop with a fresh one is not problematic and can be used to reduce errors due to drop evaporation while scanning. More critically, a variation in the height of the ring changes the drop contact area with the surface, and consequently, the DoFFI force according to eq 1 (Figures S9 and S10).

#### Scan Direction

We observe that the friction force values depend slightly on the scan direction. For the reverse scan, we measure ≈3% higher force on POS and ≈11% higher force for OTS compared to the forward scan. We attribute these differences to the presence of a force sensor, that is, a glass capillary and a ring, which creates an additional normal force on the drop.^34^ Possibly, this arrangement creates different bending moments on the force sensor for different scan directions.

From the discussion presented in this section, we conclude that this technique can be treated as a qualitative measure as it depends on the (i) drop volume, (ii) surface-to-ring distance, and (iii) the direction of scan. Thus, varying these parameters between measurements allows only a qualitative analysis of friction force values. Hence, proper adjustment of each parameter is required to make this technique quantitative.

#### Complex Patterns and Resolution

**Figure 5:**
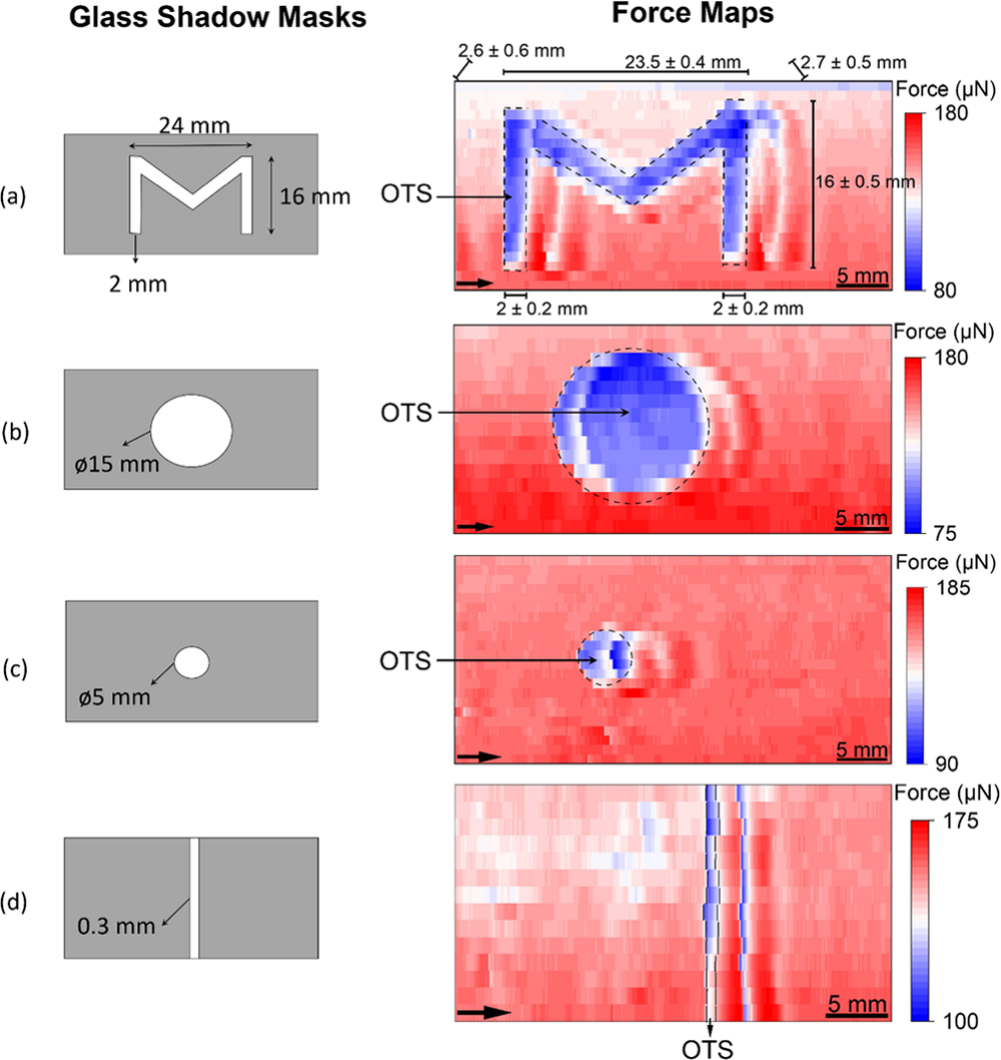
(a–d) Glass shadow masks (on the left) with dimensions and forward sDoFFI results (right) for various shaped wettability patterns. We used a volume of 15 μL for scanning. The lateral shift from scan line to scan line of the drop is 1 mm for (a) “M” pattern and (c) small circular shape and 1.5 mm for (b) large circular shape and (d) narrow OTS region. The black arrow in each force map indicates the apparent sliding direction of the drop (fast scan direction). The blue-red color scale indicates the magnitude of the friction force.

To evaluate the imaging capabilities of sDoFFI and its resolution, we create different shadow masks (Figure 5, left). The openings of the shadow masks generate a corresponding OTS layer on the glass surface. During scanning with a 15 μL drop, the drop length in the POS region is ≈4 mm and that in the OTS region is ≈3 mm. Both values are larger than the dimensions of the “M” pattern (Figure 5a), small circular (Figure 5c), and narrow OTS strip (Figure 5d) features.

For feature sizes larger than the drop length, sliding of the advancing contact line from POS to OTS (Figure 3f, v) and OTS to POS (Figure 3c, ii) is clearly visible and is characterized by a linear decrease and increase in force values, respectively. For feature size ≤ drop length, for instance, the size of “M”’s legs (≈2 mm) is half of the drop length, the advancing contact line pins at the OTS|POS transition (Figure 3c, ii) while the receding contact line pins at the POS|OTS transition (Figure 3f, v). This case constitutes increasing force values which are greater than the mean friction force value in the POS region. Therefore, we employ “50% force cutoff” criteria to identify locations of the wetting transitions and compute dimensions of the complex patterns from the wetting force maps. The cutoff force *F*_cutoff_ = (*F*_10pt_ + *F*_min_)/2 is used to generate binarized wetting maps (Supporting Information, Section 12). With these binarized maps, dimensions of the patterns are computed. *F*_10pt_ is the average of first 10 force values along a scan line and *F*_min_ is the minimum force value along the respective scan line.

First, we discuss the sDoFFI map of the M feature (Figure 5a). As discussed in part A, the pinning and depinning effects of the advancing or receding contact line render the appearance of double structures on the force map (Figure 5a, right). Even without invoking the cutoff force criteria, the M structure can be seen. However, with the criteria, we reproduce the M shape (dashed lines). We estimate the width of M’s legs from the binarized wetting map (Figure S14a) to be around 2 ± 0.2 mm. The “*V* (inclined part)” of the M structure is scanned at an angle close to 45° and we estimate the thickness of “*V*” to be 2.6 ± 0.6 mm and 2.7 ± 0.5 mm on the left and right sides, respectively. The slightly increased thickness of “*V*” arises from the interaction of the three-phase contact line with an inclined transition line. Finally, we estimate the total width of the “M” feature to be 23.5 ± 0.4 mm with a height of 16 ± 0.5 mm. With our criteria, the measured size of the M feature matches with the size of the openings of the glass shadow mask.

Second, we scan a circular shape with a diameter of 15 mm (Figure 5b, left). At the POS|OTS transition, the friction force decreases due to the slip of the advancing contact line and following the local maximum in force results in a crescent moon-like appearance (Figure 5b, right). With our criteria, we obtain a binarized wetting map (Figure S14b) and compute a diameter of 15.5 ± 0.4 mm, which is within 3% of the intended mask opening.

Third, we study a circular shape with a diameter of 5 mm on the shadow mask (Figure 5c). This feature size is close to the drop length. The edge of the OTS circle from which the drop is approached is well reproduced. Applying the cutoff criteria leads to an estimate of 5.6 ± 0.7 mm diameter.

Finally, we discuss the force signatures on the forward map of the 0.3 mm narrow OTS strip (Figure 5d). The line appears twice at locations separated by 4 mm. This distance corresponds to the drop length. With the information of the scan direction in hand, we locate the OTS|POS interline and avoid ambiguity emanating from the presence of double structures. With our criteria, we locate the positions of the POS|OTS and OTS|POS interlines and estimate the average width of the OTS layer to be 0.4 ± 0.2 mm. With these experiments, we demonstrate that an inhomogeneity having feature sizes nearly an order of magnitude less than the drop diameter can be located and resolved.

To increase the lateral resolution of the wetting map, micrometer-sized drops^18,30^ can be used. For instance, these microdrops will be very useful for the case when multiple point defects—having dimensions in the order of micrometer—are situated very close to each other. In such a scenario, it is beneficial to have an isolated signal from the corresponding individual defect to resolve them. However, for smaller water drops, evaporation decreases the width of the drop within a scan line. Thus, with these microdrops, only smaller areas can be investigated. To reduce evaporation, one can use micro-sized drops of low vapor pressure liquids, or use a humidity control chamber, or larger drops with the ring capillary configuration. We report experiments with a water drop of 15 μL volume. Water is the most commonly used liquid for wetting studies and it is nonhazardous. In addition, we are able to scan an area of 75 × 25 mm^2^ without a humidity control chamber.

We believe that the ultimate limit for sDoFFI will be the use of nano- or femtoliter drops, which are immobilized by scanning force microscopy techniques.^18^

**Figure 6:**
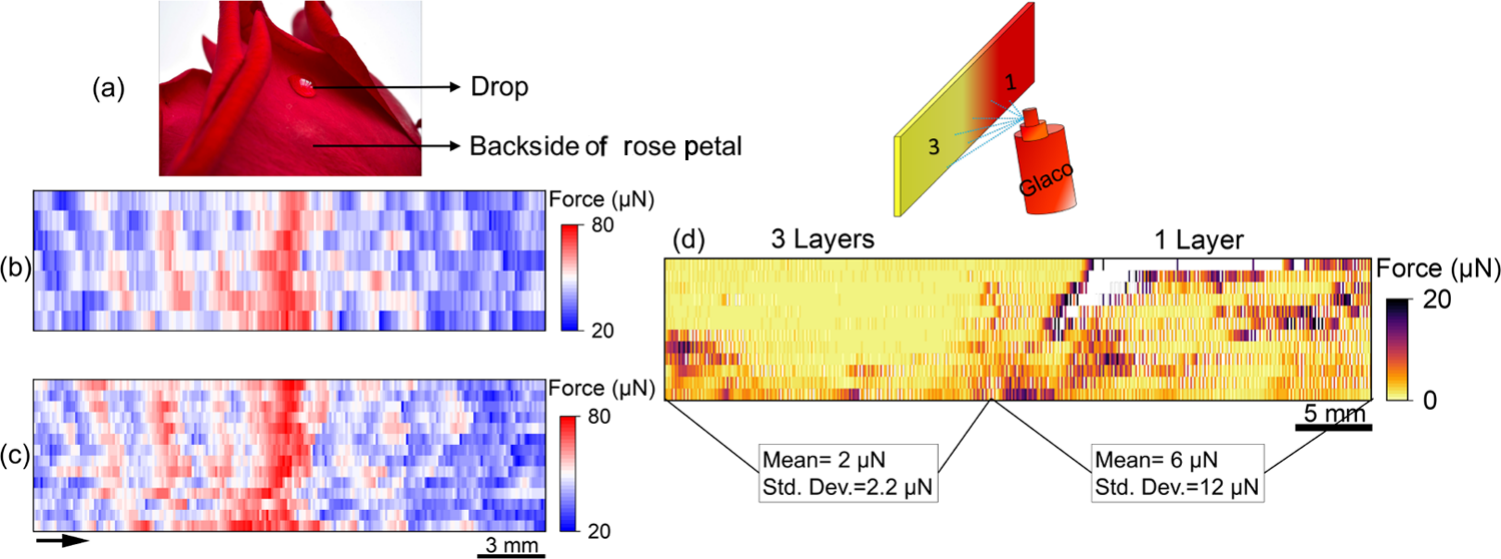
(a) A photo of a sessile water drop on a rose petal. sDoFFI is performed orthogonal to the central vein. (b) Force map; a single 2 μL water drop is immobilized by the glass capillary without the ring. The stage is moved at a speed of 3 mm/s. Here, we use an incremental lateral shift of 1 mm (slow scan axis). (c) Force map; at the start of each scan line, a fresh 2 μL drop is taken. The incremental lateral shift is reduced to 0.5 mm, corresponding to doubling of the number of scan lines. (d) Force map for a sample having two different layers of Glaco coating. A 15 μL water drop is used with an incremental lateral shift of 1 mm. By reducing the upper bound of the force scale bar to 20 μN, strong pinning sites in the force map appear as white spots in the wetting force map.

To further demonstrate the potential of the sDoFFI to scan water-repellent surfaces, we studied the backside/outerside of a rose petal and a commercial Glaco coating (Figure 6). Both samples feature unknown wetting variations. Rose petals exhibit a high static CA (>150°) with areas of high and low CA hysteresis (Figure 6a).^35^ We collected a rose petal from the garden located on our campus and studied its backside by scanning an area of 30 mm × 8 mm about 20 mm away from its receptacle (detailed preparation in Section 13, Supporting Information). The areas of low and high friction forces are clearly detectable (Figure 6b). To increase the line resolution, we used a fresh water drop at the start of each scan line, which mitigates any possible influence of evaporation on friction forces. In addition, we increased the number of scan lines for the same scanning area. Upon incorporating these two changes in the procedure, the obtained force map articulates in detail the areas of high and low adhesion and elucidates the ridge structures present on the backside of the petal (Figure 6c).

Spray coatings made by Glaco are used for coating the windshield of cars so that rainwater does not stick to the surface, improving visibility. We used the commercially available superhydrophobic coating (Soft Glaco mirror coat zero) to prepare a sample having one half coated with three layers and the other half coated with one layer. A liquid suspension is sprayed onto the glass substrate from nearly 15–20 cm away. The surface that we obtained by spraying one layer, that is, on the right side of the sample, exhibits several scattered areas of friction forces (brown areas in Figure 6d) and one area at the edge where the friction force values exceeded 20 μN (white areas in Figure 6d). During the coating preparation, we noticed that on the right half, the final Glaco liquid evaporated on the top right of the sample. After the coating procedure, no visual differences between the two sections are observable. In contrast to the single layer of Glaco, the sDoFFI force map reveals a more uniform area on the three-layer side. The areas exceeding the friction force value of 20 μN appear white. However, sparse areas exhibiting lateral forces up to 10 μN are detected (Figure 6d and Supporting Information, Section 14). Hence, we observe that spraying three layers of Glaco results in a more homogeneous surface. However, the coating uniformity is also influenced by the operator skill. Overall, sDoFFI can be used for process control and for optimizing the application process of technical coatings.

In the following example, we prove that with sDoFFI, we can study the differences in wetting properties arising from degradation of the surface due to sliding drops. Such sliding drop experiments are omnipresent. Drops sliding along a surface may result in wear, abrasion, or adaptation of the surface.^36,37^ These effects lead to changes in the surface properties but the associated chemical and topographic variations are not easily locatable. In particular, wear, abrasion, or adaptation may depend on the sliding drop number and its distance from the drop impact position. To map the wettability changes and trajectory followed by the sliding drop, we perform sDoFFI.

**Figure 7:**
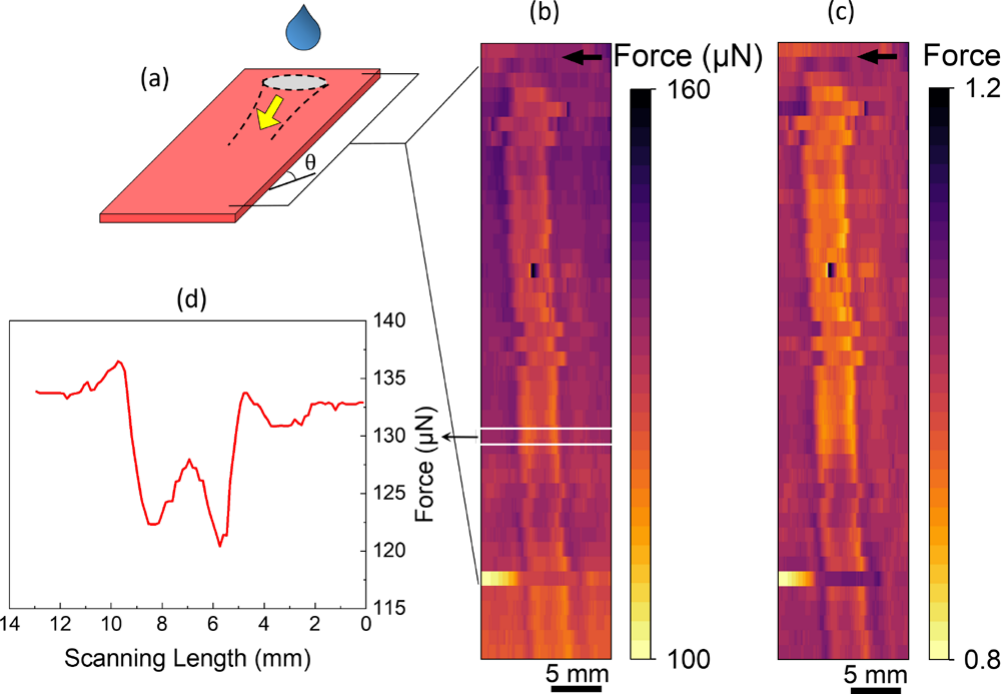
(a) Schematic representing the drop wear experiment. 5000 drops, 33 μL each, of ultrapure water (Milli-Q) are run down a single path on the PFOTS-coated glass sample at a tilt angle of 50°. The drops are deposited from a height of 5 mm at the starting point with 1.5 s time interval between each drop. (b) sDoFFI map of the sample using a drop with a volume of 15 μL. The fast scan axis is parallel to the width of the sample and the slow scan axis is advanced by 1.5 mm. The apparent drop motion is indicated by a black arrow above the map. (c) Normalized force map to compensate the effect of evaporation. The mean of 10 force data points at the start of each scan line is considered for the normalization of the respective scan line. (d) Force profile along with one of the scan lines corresponding to the force map in panel (b).

We let 5000 drops of Milli-Q water to run down a single path on glass coated with PFOTS (Figure 7a). A visual inspection by eye or by an optical microscope of this sample does not reveal any contrast between the trajectory area and the area outside. With sDoFFI, we are able to map the drop impact area and the path paved by drops while descending (Figure 7b).^38^ The sDoFFI measurement indicates a lower sliding force for areas on the pathway (122 μN) compared to areas outside of this path (135 μN). To compensate for the drop evaporation effects, we normalize the force values along each scan line with the average of a few force values obtained just after the start of drop motion (Figure 7c). Furthermore, the sliding force for each scan line is slightly higher before entering the rundown area, followed by a local minimum in force (Figure 7d). Such signatures result from the pinning of the drop’s advancing and receding sides at the right and left edges of the path as observed for the model having a sharp transition in wetting properties.

The purpose of these final experiments (Figures 6 and 7) is to demonstrate that the sDoFFI is able to record spatially varying wettability information. The location of edges of the entire trail along the path would be nearly impossible, or at least very time-consuming, by standard CA goniometry or by existing force-based characterization methods. This sDoFFI experiment revealed nearly 4000 force values on an area of 62 × 20 mm^2^ in approximately 6–8 min. Unraveling the details of the wetting behavior at a submillimeter scale provides insights on the rose petal, Glaco coat (Figure 6), and of surface wear due to the sliding drop (Figure 7). Hence, our experiments with the water drop did not reach the limit of lateral resolution yet. Thus, the sDoFFI technique is advantageous for mapping varying wetting properties of surfaces caused by either chemical or topographic heterogeneity.

## Conclusions

Stick–slip phenomena have been observed in drop sliding experiments on patterned hydrophilic and hydrophobic surfaces using tilted plate setups.^39−42^ Drops sliding down the tilted plane always slide under the influence of body force, which may result in missing insights into the stick–slip phenomenon at the transition line. However, sDoFFI provides us with the control of the drop’s position and velocity, thereby allowing us to fetch intricate details of the drop’s stick–slip behavior. Studying the example of an OTS|POS sample with sDoFFI, we elucidate the details of the friction force that acts on a drop. In particular, the sliding directed toward a high CAH area reveals two force maxima. The process follows a stick–slip–stick–slip sliding behavior with global maxima in force for the second stick process, which is imparted by the receding contact line. In the reverse direction, that is, sliding toward the surface with a lower CA hysteresis, the drop slips, sticks, and slips again. In this case, a local maximum in force is detected. This local maximum in sliding force has to be overcome for drops that are sessile on top of the transition line. Drops that slide faster may have enough momentum to pass the interline from higher to lower CA hysteresis without realizing this local maxima.

The sDoFFI is a new tool for 2D characterization and imaging of surface wetting properties. The force signal arising from the interaction of the three-phase contact line with the inhomogeneities is used to locate and resolve the wetting features from centimeter to submillimeter sizes. Even surface features having sizes much smaller than the drop diameter can be characterized. Thus, sDoFFI is not limited to laboratory-based samples but also characterizes biological and commercial surfaces. The outcome of the rose petal and Glaco scanning highlights the wide scope of the technique, which holds potential in process control and optimization.

## Supporting Information Available

Discussion on CAs, ring-to-surface distance, drop parameters, and supporting figures (PDF)
Video showing how to use sDoFFI (MP4)
