# Supplementary material for: Scanning Drop Friction Force Microscopy
Source: Langmuir. 2022 Nov 18;38(48):14635–43. doi: 10.1021/acs.langmuir.2c02046 (PMC9730904; doi:10.1021/acs.langmuir.2c02046)
Supplement: Supplementary file 1 — la2c02046_si_001.pdf [file la2c02046_si_001.pdf]

Supporting Information

Scanning Drop Friction Force Microscopy

Chirag Hinduja[1], Alexandre Laroche[1,2], Sajjad Shumaly[1], Yujiao Wang [3,4], Doris Vollmer[1], Hans-Jürgen Butt[1], Rüdiger Berger[1*]

[1] Max Planck Institute for Polymer Research, 55128 Mainz, Germany.

[2] University of Zurich, Winterthurerstrasse 190, 8057 Zurich, Switzerland.

[3] Key Laboratory of Interfacial Physics and Technology, Shanghai Institute of Applied Physics, Chinese Academy of Sciences, Shanghai 201800, China.

[4] University of Chinese Academy of Sciences, Beijing 100049, China.

[*] Corresponding Author's E-mail: berger@mpip-mainz.mpg.de

1. **CAs variation on the two areas**:

Sessile Drop Method:

To characterize the hydrophobicity of the samples, contact angle goniometry is performed prior to the sliding drop experiments. Fig. S1 shows the corresponding variations in advancing and receding CAs on the respective area on a sample. Krüss DSA 100 goniometer is used for the measurements. Measurements are done at 3 different locations.

Drop sliding CAs:

Fig.S1 e,f shows CA hysteresis on different POS|OTS samples. The samples are prepared on different dates and months of the year. We observe a significant variation for the receding CAs across the samples. Error bars represents variation of ±2σ. We attribute this difference to the change in surface wetting properties across the samples.

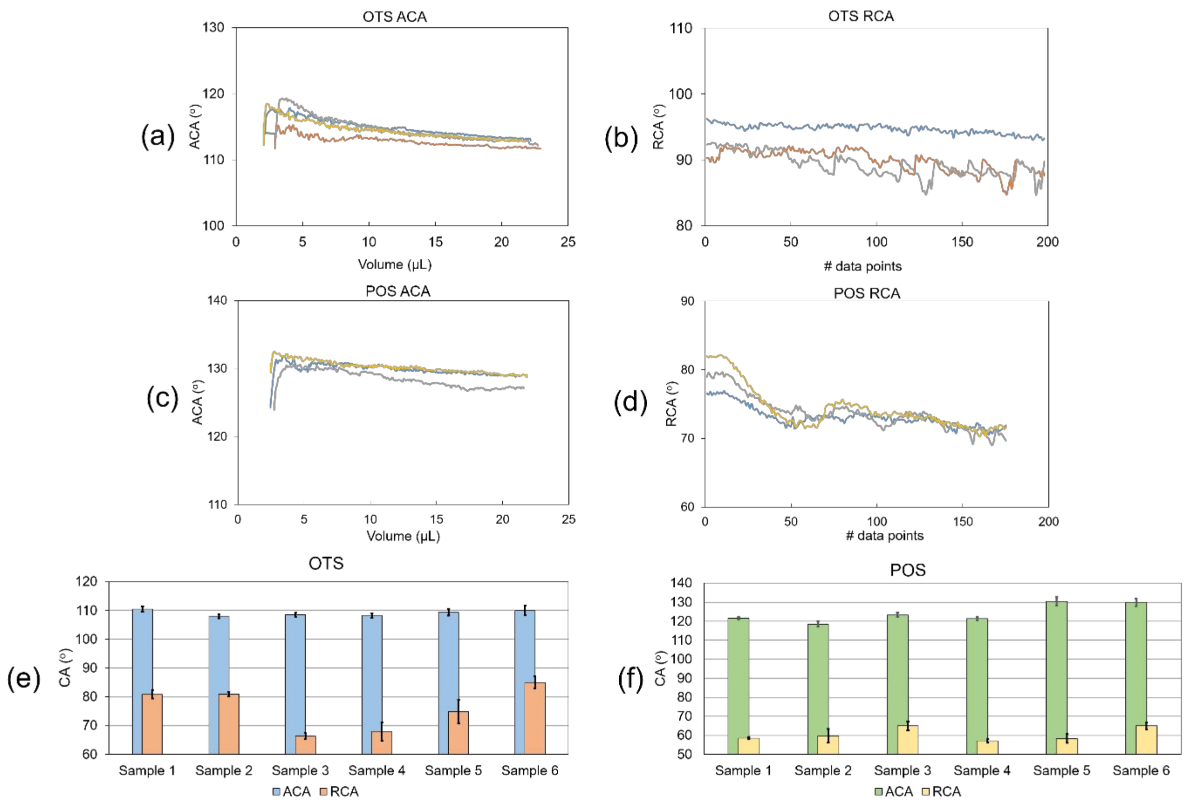

Figure S1. Contact angles measured from sessile drop method for (a,c) OTS side, and (b,d) POS side. CAs during drop sliding (sDoFFI) on different POS|OTS samples. (e) CAs on OTS side, (f) CAs on POS side.

2. **Sensor Calibration**: As the primary method for sensor calibration, the end of the capillary is given a gentle push and is allowed to undergo free damped oscillation (Fig S2). During oscillation, the amplitude of deflection of the capillary is recorded via a CMOS camera to determine the fundamental frequency of vibration (ω_n). The obtained frequency data is used to calculate the spring constant of the capillary using Equation 2.

In the second method for calibration of the spring constant of the capillary, the free end of the capillary is displaced by a known amount. Corresponding to this deflection, force values obtained by a weighing balance are recorded (Inset Fig S3). The slope of a linear regression between load and deflection corresponds to the spring constant (Fig. S3). The relative difference in the spring constant determined by these two methods is less than 3 %.

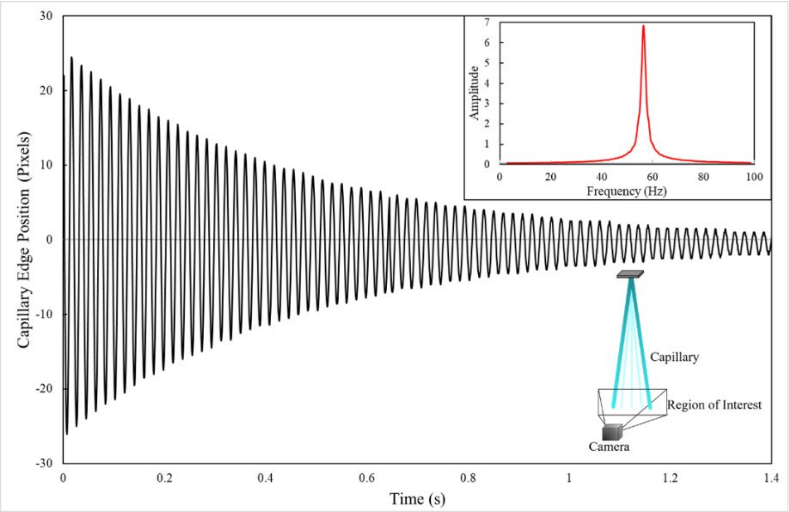

Figure S2. Dynamic calibration: Vibrations of the edge position at the apex of the capillary. Insets include a Fourier-transform plot of the waveform and a schematic showing the procedure for determining ω_n.

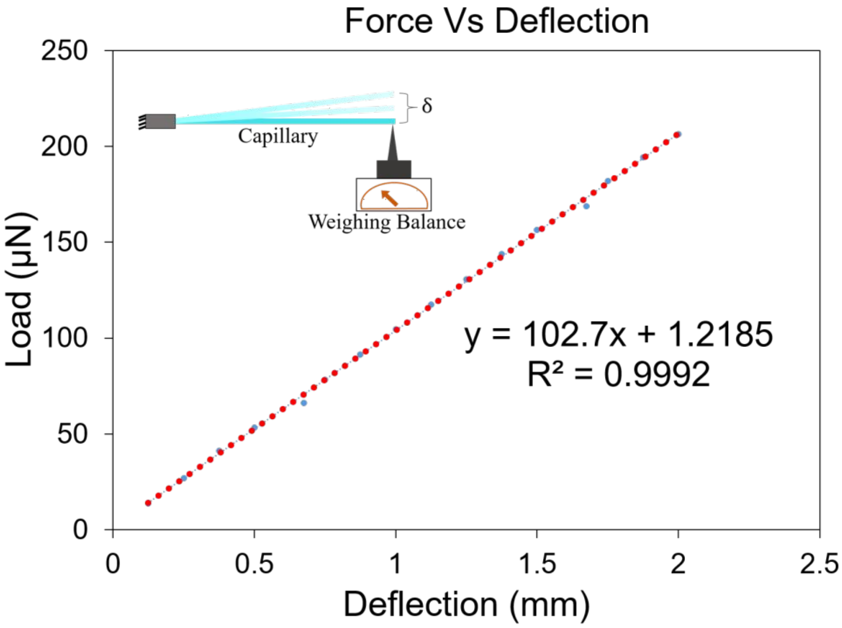

Figure S3. Applied Load-Deflection calibration: The inset shows a schematic of measurement procedure.

3. **Generating Wetting Force Maps**: We relate the temporal evolution of each frame to corresponding spatial coordinates ($x_{i,j}$) on the sample by the video recording speed ($t_{video} =$

30 fps) and the speed of the stage (v_{stage}); $x_{i,j} = \left(v_{stage} \cdot \frac{1}{t_{video}}\right) \cdot j$. Here, '$i$' corresponds to the number of the scan lines and 'j' to the count of the frame.

Any friction force that the drop experiences result in a deflection of the capillary. This deflection (δ) is measured optically using a side view CMOS camera (Krüss DSA 100) (Fig. 2c). The friction force is calculated by $F_{DoFFI@i,j} = \kappa \cdot \delta_{i,j}$. This force is plotted for all positions in the form of force maps. The areas where the XY-stage stops, changes its moving direction, or shifts to the subsequent scan line are discarded from images.

4. Determining kinetic regime beginning on the force curve: In figure 3 (a, b), dotted area shows the force values which are not considered for the wetting maps. This dotted area comprises of static and transition regime on the force curve. The transition from a static to kinetic regime could range up to several millimeter (reference 21, Nan Gao *et al.*). We experimentally measure length of the static regime and transition regime (fig.S4). In our cases, the length of the static and transition regime corresponds to ≈ 5 mm. To be on the safe side, we consider force data after the stage has moved by ≈ 6 mm in the forward wetting map and ≈ 7 mm in reverse wetting map. This corresponds to the starting of the plateau -adjacent to the static and transition dome. We select this starting point as the zero position on our map.

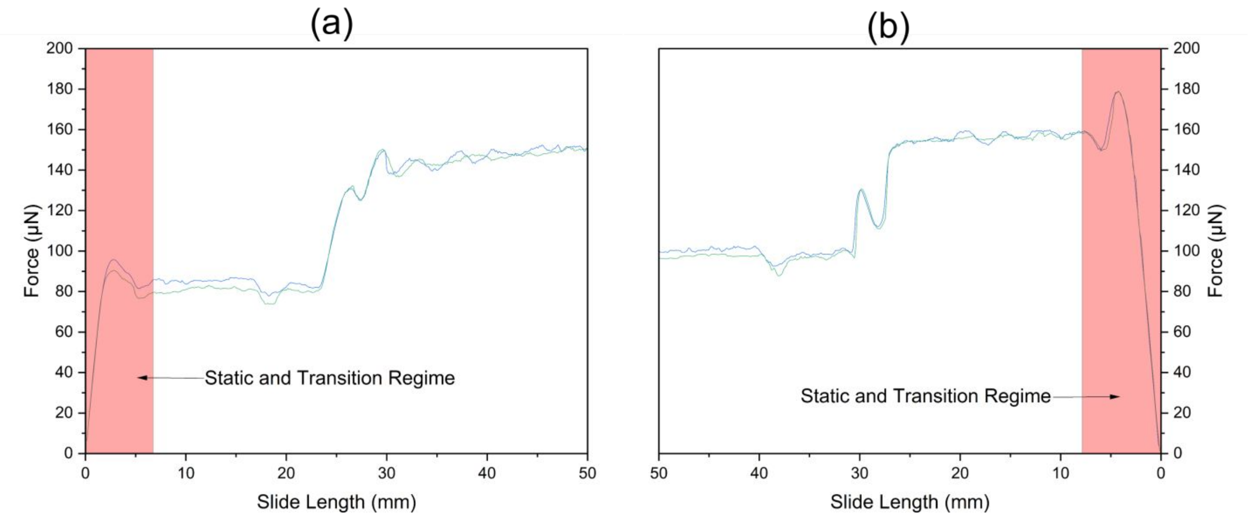

Figure S4. The force profiles of a 15µL drop for 2 scan lines. For generating wetting force maps, the force data corresponding to static regime has been discarded. The data next to this red region in (a) forward motion, (b) reverse motion is considered for characterization.

5. Drop length variation along a scan line: Fig. S5 shows the drop length variation along a scan line. The peak in region (iii, Figure 3c) has the same magnitude than uncertainty of the measurement. However, this peak is not associated to uncertainty of the measurement as it appears in each scan line always at the same position (plotted in grey in Figure 3c). This peak corresponds to the time when receding CL apex is sticking to the inter-line. We confirm that the 2nd peak is out of the uncertainty by analyzing the dynamic CAs (fig 3(e-iii)). We measured an increase in advancing CA and a slight decrease in receding CA, which suggests that whole droplet inclines more to the direction of motion. The latter corresponds to an increasing in friction force. Also, the analysis of drop length (Fig S5) shows an increase in drop length when the receding CL interacts with the inter-line.

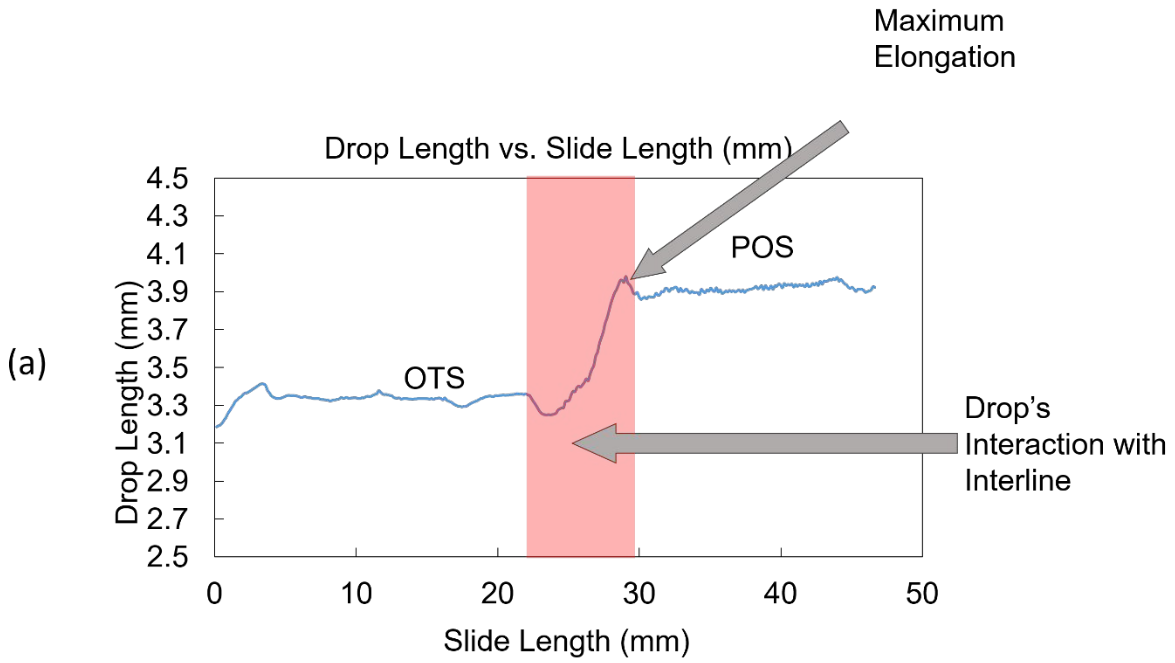

Figure S5. Drop length variation when it slides from OTS to POS region.

6. Width and contact area on POS and OTS side: We calculate width of the 15µL drop along a scan line. It is 2.9 ± 0.15 mm on OTS surface and 2.1 ± 0.1 mm on POS (Fig. S6). In addition, we estimate the contact area of the sessile drop in OTS and POS region. A 15 µL drop is deposited via micropipette on each side. Subsequently, we determine drop base diameter. Drops at 6 different locations on each side are considered for base diameter averaging. We assume

circular base area for the sessile drop and calculate the area of this circular foot print i.e., $\frac{\pi}{4}D_{base}^2$. For the OTS side we estimate area to be 8.7 ± 0.05 mm^2 and for POS side, 7.5 ± 0.15 mm^2.

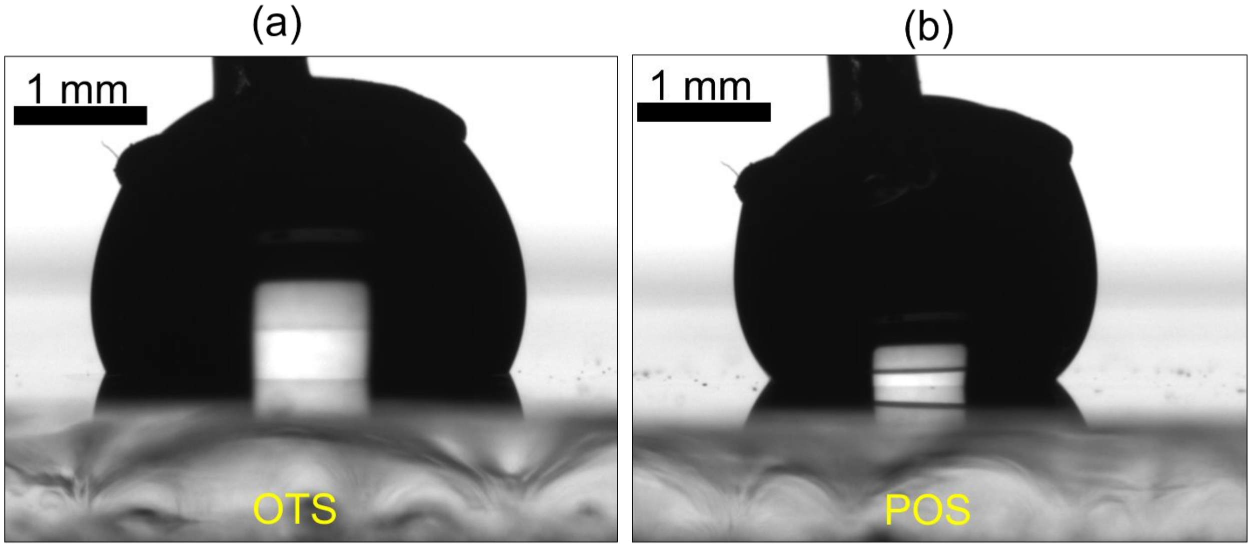

Figure S6. Front view of a 15 µL drop on (a) OTS side, (b) POS side.

7. Three phase contact line shape: To determine the shape of three-phase CL, we image a 6µL drop from the bottom side when it slides on the OTS and POS region. We use the reflective imaging system to acquire the actual shape of three-phase CL to avoid any ambiguity arising out of the drop overhang. Fig. S7 shows the bottom image when a drop slides (at 1mm/s) on the (a) OTS and (b) POS area. It is evident from the figure that the drop foot print is circular/Oval in shape for the OTS side and, on the POS side, it is elliptical.

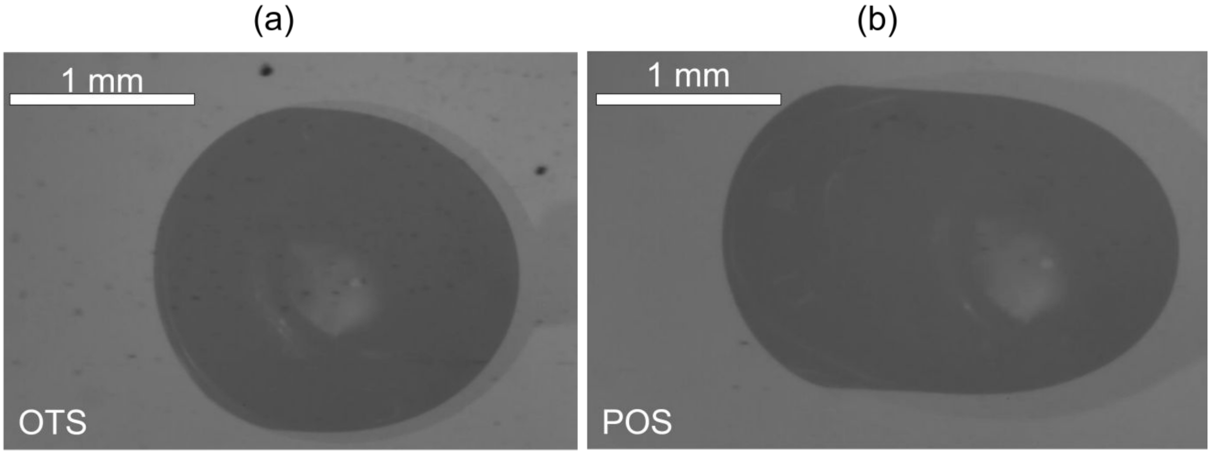

Figure S7. Three-phase CL image of the 6µL drop when it slides on (a) OTS side and (b) POS side.

8. **Drop evaporation between first and last scan-line.** As the drop evaporates while scanning, the width of the drop reduces continuously from the first to the last scan line. This width decrease manifests in the form of a decrease in force values (according to Equation 1). Both Fig.S8 (a) and (b) show the decrease in force values between the first and the last scan lines.

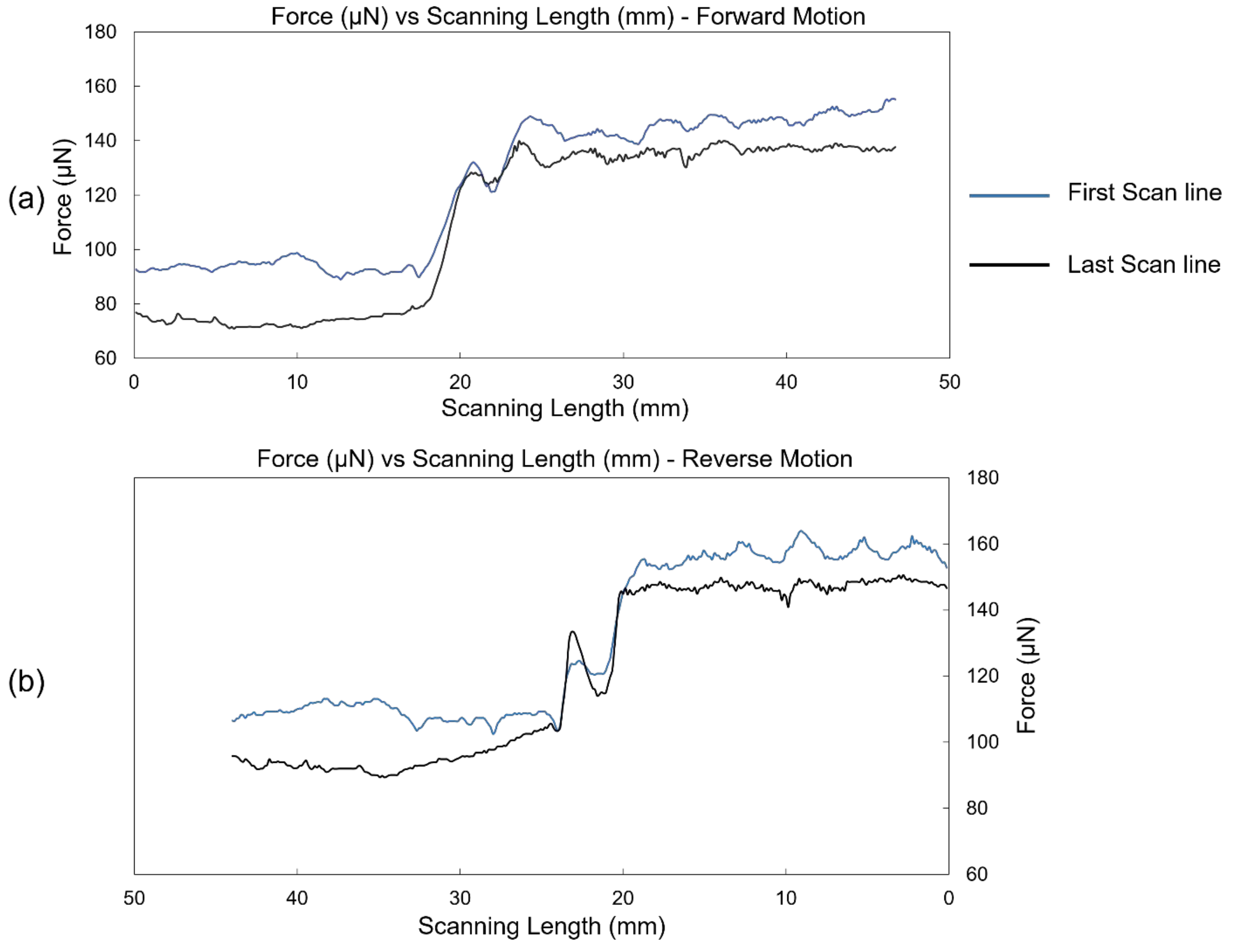

Figure S8. For the OTS|POS sample: (a) Forward force profiles for first and last scan-line. (b) Reverse motion force profiles for first and last scan-line.

9. **Influence of ring height and stage speed**: All the experiments, in which a ring capillary is used, are performed by visually adjusting the distance of the ring from the sample such that a reasonable drop shape is retained. For a constant drop volume, an increase in ring height results in a decrease in drop footprint, and consequently a decrease in force (Fig. S9). The abscissa of Fig.S9

shows the ring to surface distance relative to height of the sessile drop. The height of sessile drop is averaged over 5 drops at 5 different locations on the sample. No significant change has been observed on dynamic contact angles for all the shown ring to surface distances (Fig. S10). As long as the height of the ring is kept constant, no observable change in force is observed between measurements using different drops. Also, no change has been observed for scanning speed variations (Fig. S11). However, a difference of 10% in force is observed between forward and reverse measurement directions.

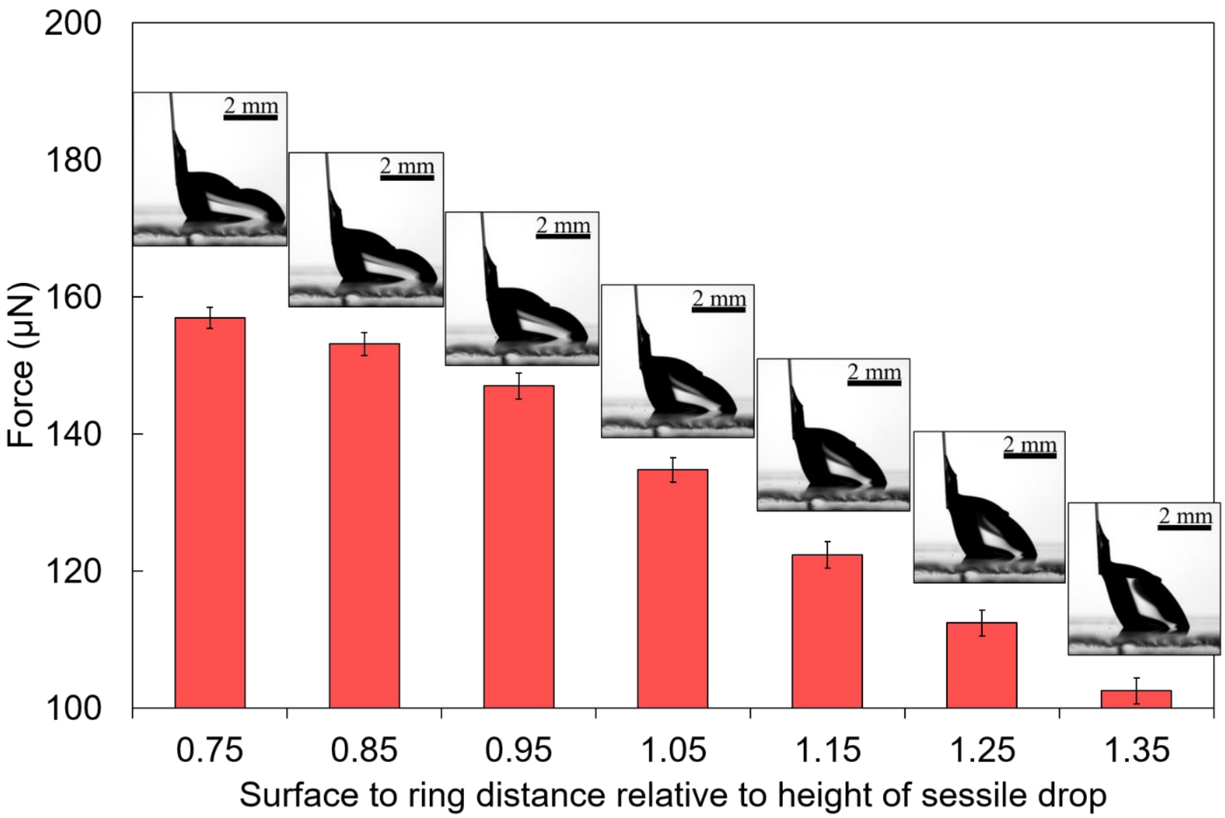

Figure S9. Influence of ring to surface height on scanning force. For this investigation, a 15µL Milli-Q water drop on glass coated with only PFOTS is considered. The smallest ring to surface distance is 1.4 ± 0.05 mm and subsequently, the ring to surface height is increased by 0.2 mm. The height of the sessile drop is 1.9 ± 0.05 mm. Here, scanning speed is kept constant at 4 mm/s.

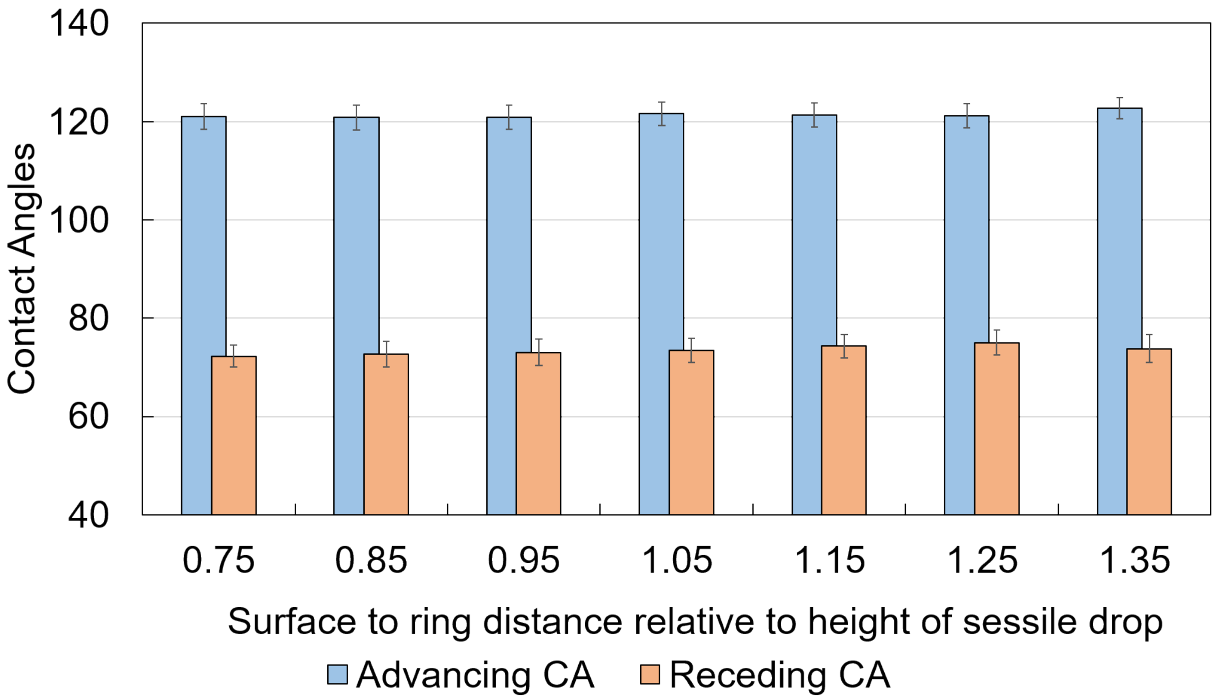

Figure S10. Dynamic contact angles of a drop sliding with different ring to surface distances. The angles have been averaged within the scan line. The data have been computed from the same set from which force data is obtained (Fig. S9).

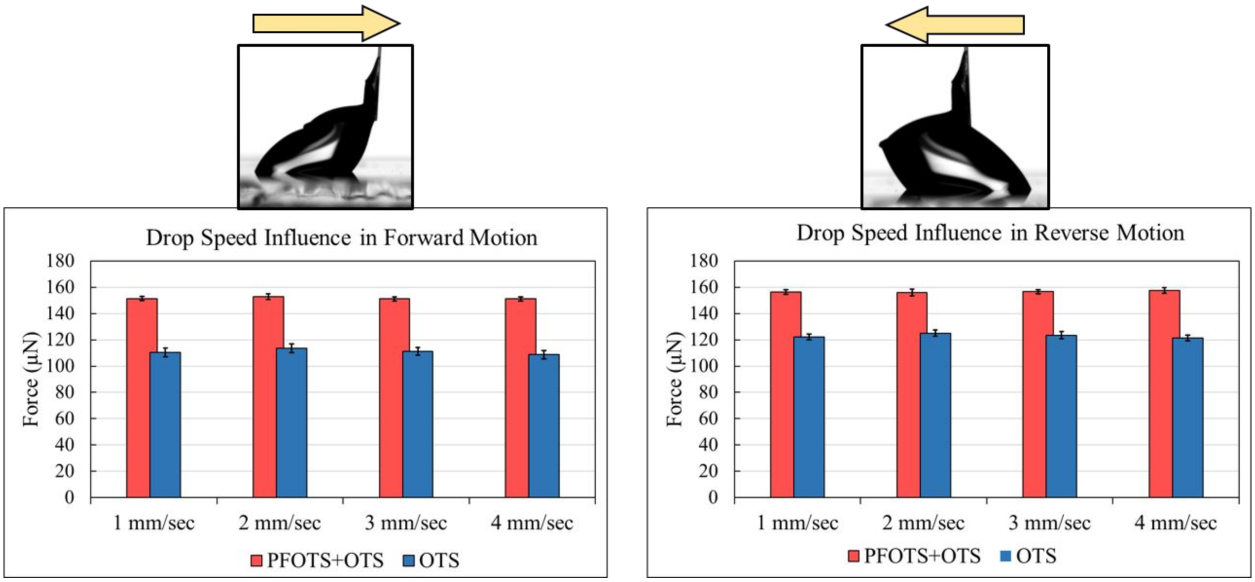

Figure S11. Influence of drop scanning speed on scanning force. For this investigation a 15 μL Milli-Q water drop on the OTS|POS sample is considered. Each data representation corresponds

to an average of 3 different water drops on 3 different scanlines on the sample. For this investigation, the ring to surface height is kept constant at 2 mm throughout.

10. Contact Angles for same ring height and different volumes: To ascertain that presence of ring doesn't alter the CA values, we performed additional experiments with 10, 15, 20, 25 µL drop volume on OTS|POS sample. We keep ring to surface height same for all the volumes. The height is decided according to the 15 µL drop. We measure the advancing and receding CAs for all the volumes and for both the directions. However, we do not see any significant change in CAs among the volumes. Therefore, we conclude that CAs are dictated by surface chemistry and topography and, presence of ring does not influence the CAs.

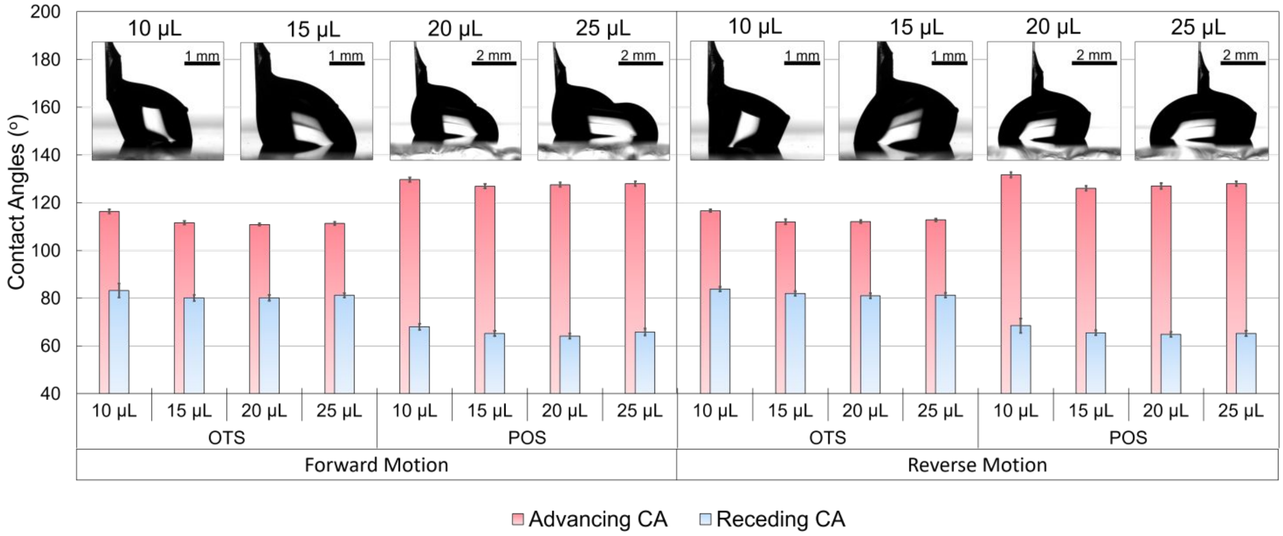

Figure S12. Contact angle variation along a scan line for different volumes. The speed of the stage is kept constant at 4mm/s. The ring height is decided according to 15 µL drop volume and kept constant for all the volumes.

11. **Reverse force map for complex geometries:** Fig. S13 shows wetting maps obtained while reverse scanning.

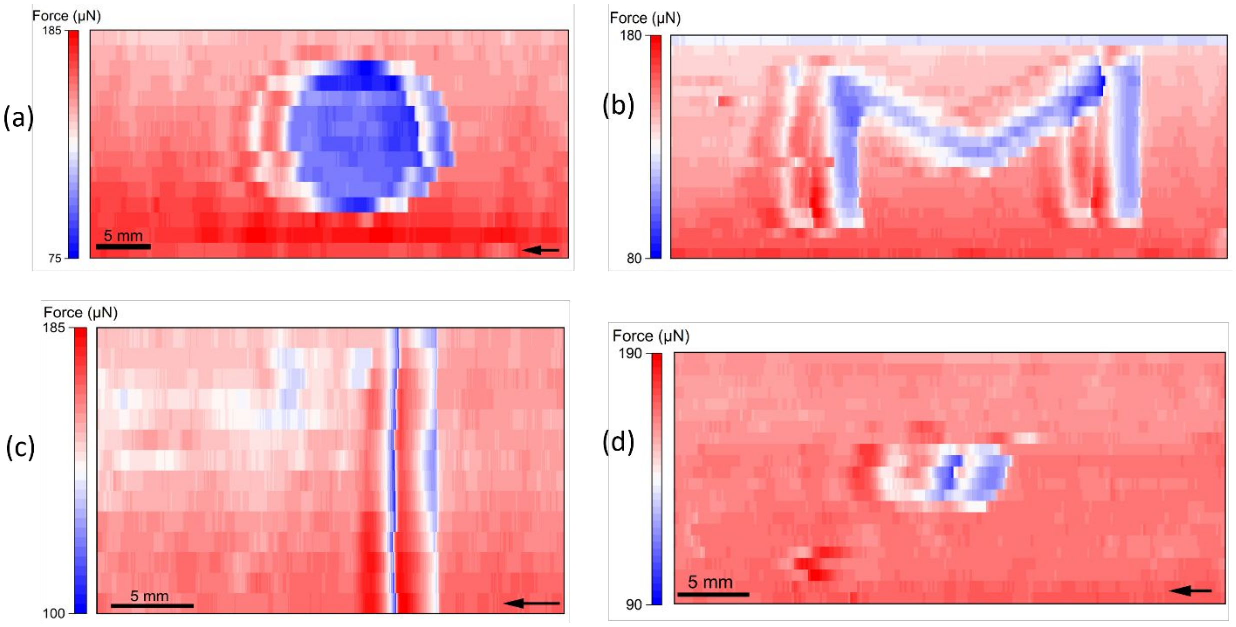

Figure S13. Reverse wetting force maps: (a) Circular shape (Diameter ≈ 15 mm), (b) M shape, (c) Narrow OTS layer (≈ 0.3 mm), (d) Small Circular (diameter ≈ 5 mm).

12. Binary wetting force maps and dimension calculations: Using the cutoff criteria described in the main text, we generate binary wetting maps for each of the complex shapes (Fig.S14). We designate force values in the map above the cutoff force as 1 and values below the cutoff value as 0. The obtained binary maps are analyzed via MATLAB script to compute their dimensions. For large and small circular shapes the diameter in the x- and y-directions, and at two 45° angles are averaged.

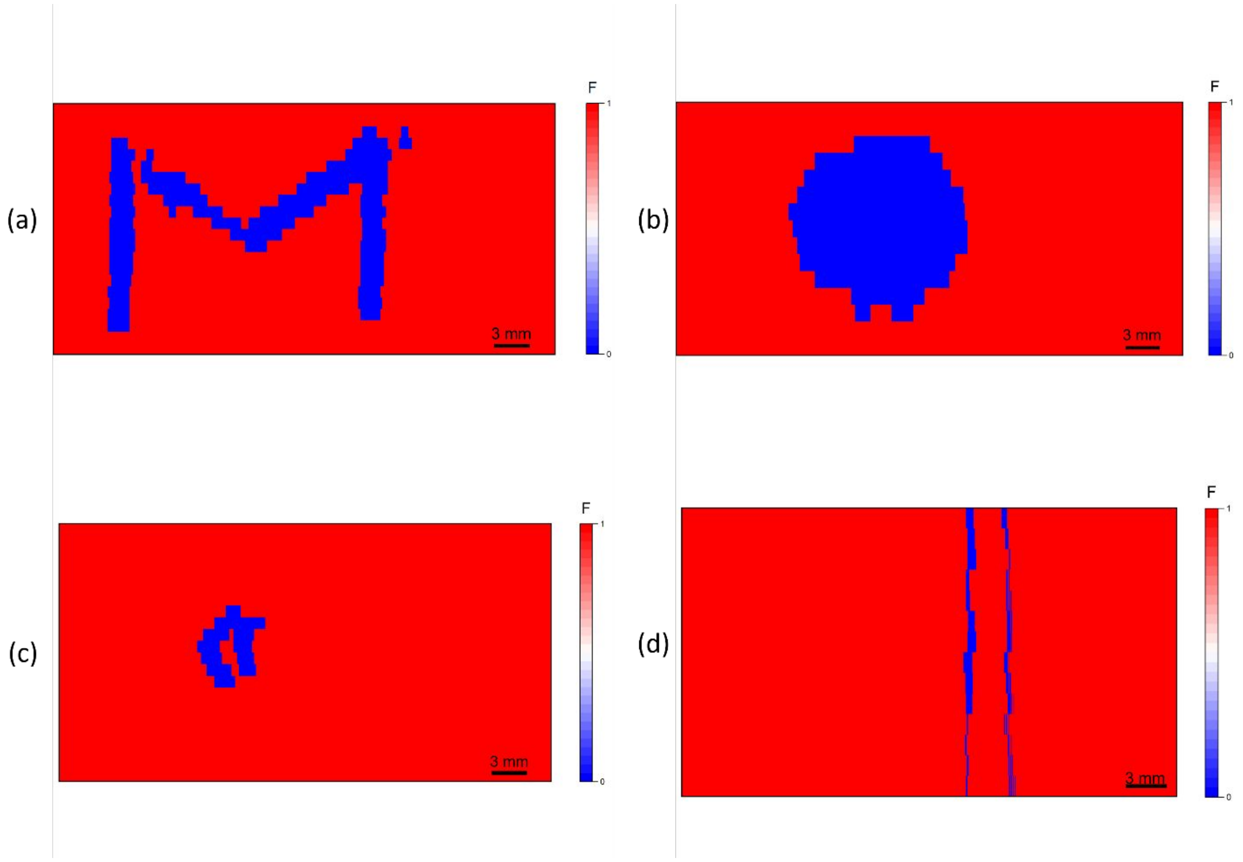

Figure S14. Binarized wettings maps: (a) M shape, (b) Large Circular shape, (c) Small Circular Shape, (d) Narrow OTS strip pattern. Blue (OTS) and red (POS).

13. Rose petal preparation: We plucked the rose petal from the garden and a fully grown petal was selected from this rose flower. We carefully take this petal and cut it in a rectangular shape, larger than the scanning area i.e., 30mm x 8mm. We were very careful in not touching the area which was intended for scanning. We chose a rectangular area in the mid of rose petal, which was relatively flat compared to peripheral part. Then, we place the cut rose petal sample on the glass slide, stick it from one end using scotch tape and then select the opposite and stretch it gently and stick the tape to the substrate. In the same fashion we cover all the four sides of the rectangle with the slices of scotch tape. We gently blow the nitrogen air through a blower to remove any physical impurity deposited on the top. We want to emphasize that, on the day of experiment, plucking rose petal and sDoFFI analysis was done within the span of 1 hour implying that rose petal remained fresh.

14. **Negative forces in Fig 6d.** The depinning of the droplet while scanning over these pinning centers causes the capillary sensor to swing back and deflect in the opposite direction. Thus for a short period of time negative friction force values are measured. These negative values have been hidden on the force scale bar by setting the lowest value on the scale to 0. For more sensitive scanning one could use capillaries having a lower spring constant.

15. **Sample rotated by 180° before reverse motion.** To inspect whether asymmetry introduced by the ring influences the appearance of stick-slip behavior, we rotate the sample by 180° after the trace (forward motion) along a scan line. In the trace motion, drop slides from OTS to POS side (Fig. S15 (a), black profile). After rotation of the sample by 180°, in the re-trace (reverse motion), drop again slides from OTS to POS along the same scan line. To avoid any volume loss, we replace the drop before embarking reverse motion. In the same way, we inspect for POS to OTS direction (Fig. S15 (b)). It is evident from the Fig. S15 that stick slip behavior is not influenced by the presence of the ring.

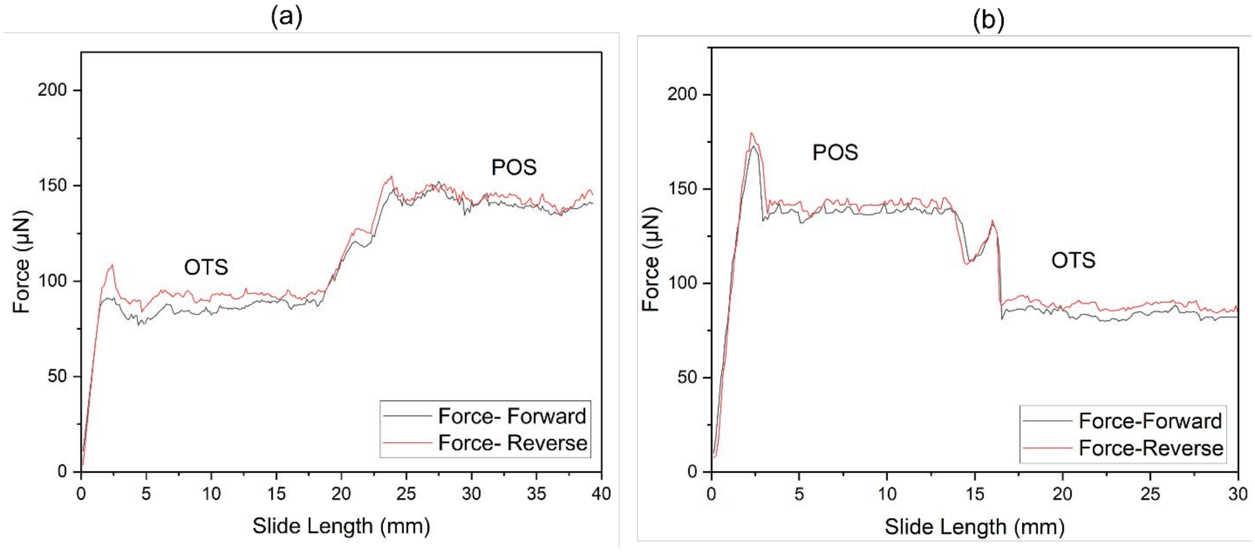

Figure S15. Force profiles along a scan-line for the case when sample is rotated by 180° before the re-trace motion. A 15µL Milli-Q water drop is used for the both trace and re-trace at 4mm/s stage speed. (a) Force profiles indicating stick-slip when drop is pulled (black profile) and pushed (red profile) from OTS to POS side. (b) Force profiles indicating stick-slip when drop is pulled (black profile) and pushed (red profile) from POS to OTS side.

16. Scanning Force Microscopy Results on OTS|POS sample: We performed scanning force microscope investi…..

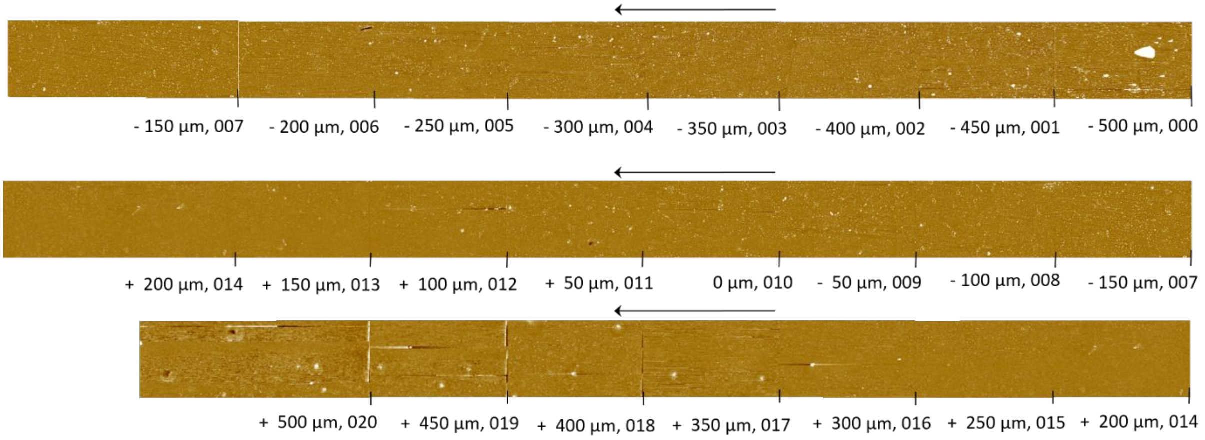

Figure S16. Scanning force microscopy images in the vicinity of POS|OTS interline.

17. **Experimental setup micrographs and volume replenishment demonstration.** Here we provide closer look to the experimental setup. Fig. S9 shows the details of the experimental setup. We have used Krüss DSA 100 machine with embedded X-Y stage. Also, we have provided the information via a video file on "how in-train drop volume replacement could be done".

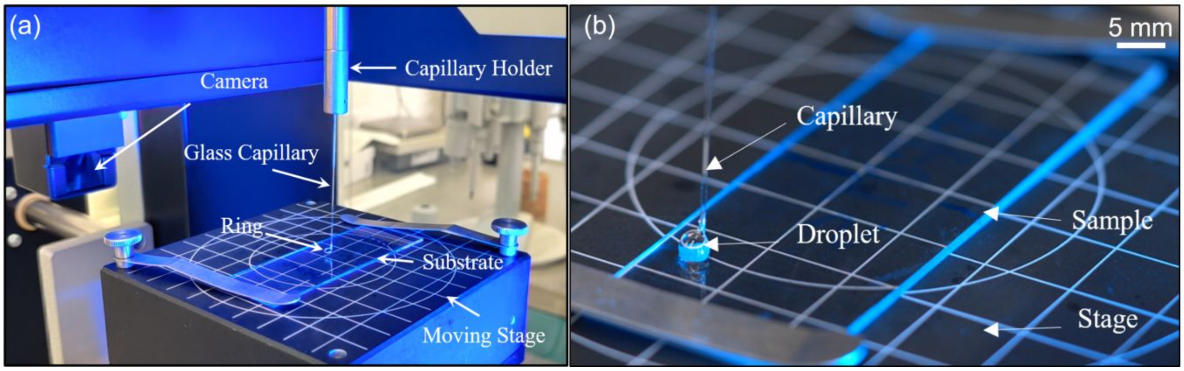

Figure S17. (a) Photograph elaborating experimental setup. (b) Close-up of the Drop-ring-capillary-surface configuration.
